# Large scale multiplex PCR improves pathogen detection by DNA microarrays

**DOI:** 10.1186/1471-2180-9-1

**Published:** 2009-01-03

**Authors:** Maria Palka-Santini, Berit E Cleven, Ludwig Eichinger, Martin Krönke, Oleg Krut

**Affiliations:** 1 Institute for Medical Microbiology, Immunology and Hygiene, Medical Faculty, University of Cologne, Germany; 2 Vulkan Technic Maschinen-Konstruktions GmbH, Germany; 3 Center for Biochemistry, Medical Faculty, University of Cologne, Germany; 4 Center for Molecular Medicine Cologne, Medical Center, University of Cologne, Germany

## Abstract

**Background:**

Medium density DNA microchips that carry a collection of probes for a broad spectrum of pathogens, have the potential to be powerful tools for simultaneous species identification, detection of virulence factors and antimicrobial resistance determinants. However, their widespread use in microbiological diagnostics is limited by the problem of low pathogen numbers in clinical specimens revealing relatively low amounts of pathogen DNA.

**Results:**

To increase the detection power of a fluorescence-based prototype-microarray designed to identify pathogenic microorganisms involved in sepsis, we propose a large scale multiplex PCR (LSplex PCR) for amplification of several dozens of gene-segments of 9 pathogenic species. This protocol employs a large set of primer pairs, potentially able to amplify 800 different gene segments that correspond to the capture probes spotted on the microarray. The LSplex protocol is shown to selectively amplify only the gene segments corresponding to the specific pathogen present in the analyte. Application of LSplex increases the microarray detection of target templates by a factor of 100 to 1000.

**Conclusion:**

Our data provide a proof of principle for the improvement of detection of pathogen DNA by microarray hybridization by using LSplex PCR.

## Background

Clinical microbiological diagnostics, environmental survey, food quality control and biodefence strategies have a common keystone: accurate and rapid identification of pathogenic microorganisms. Several molecular biology-based methods have been recently developed for microbial diagnostics and offer noticeable advantages over conventional techniques in microbiology. Among the molecular biology-based methods, DNA microarray technology presents the potential of direct and rapid identification of multiple DNA sequences [1-7]. A microarray displaying DNA probes corresponding to a collection of genes of a broad spectrum of pathogens is a powerful tool for simultaneous species identification, detection of virulence factors and antimicrobial resistance determinants [2]. Major drawbacks in using DNA microarrays as a standard technique for pathogen detection are linked to the low representation of pathogen DNA in the analytes, but also to the relatively low sensitivity of fluorescence-based microarrays. The amount of specific pathogen DNA present in clinical, environmental, and food samples is sometimes as low as few femtograms [8-14], while the detection limit for genomic DNA in fluorescence-based microarrays, without any pre-amplification, is in the range of micrograms to nanograms [1,3,4,7,15].

A solution to overcome this intrinsic weakness of fluorescence-based microarrays is to specifically amplify the pathogen DNA fraction in the sample in order to increase the sensitivity level of detection. The question of random or selective pathogen DNA amplification prior to DNA microarray detection has been already addressed [16] and applications of multiplex PCR using a small number of primer pairs corresponding to the capture probes on low density microarrays have been published [16,5,6,16-18]. We present here a further development of this approach, by proposing a large scale multiplex PCR adapted to the format of a prototype medium density microarray developed in our laboratory, employing up to 800 specific primer pairs. The limiting conditions for the LSplex PCR protocol are empirically determined and the resulting amplification biases are evaluated.

## Methods

### Strains of microorganisms used for the preparation of DNA templates

Template DNA was prepared from the following bacterial and fungal reference strains, obtained from the American Type Culture Collection (ATCC, Manassas, Va.), the Deutsche Sammlung von Mikroorganismen und Zellkulturen (DSMZ, Braunschweig, Germany) or the Collection de l'Institut Pasteur, (CIP, Paris, France): *Staphylococcus aureus *(ATCC 29213 and CIP 65.6), *Staphylococcus epidermidis *(ATCC 12228), *Escherichia coli *(ATCC 25922 and CIP 105893), *Pseudomonas aeruginosa *(ATCC 27853 and CIP 105765), *Klebsiella pneumoniae *(DSM 681), *Proteus mirabilis *(DSM 788), *Enterococcus faecalis *(ATCC 29212), *Streptococcus pneumoniae *(CIP 106577), *Streptococcus mitis *(CIP 104997), *Candida albicans *(ATCC 10231). A clinical isolate of *S. aureus *(T100) was also used in some experiments. Microorganisms were grown over night at 37°C with constant shaking at 220 rpm in 5 ml Luria-Bertani (LB) broth or tryptic soy broth (TSB, 30 g/l, Merck) containing 3 g/l yeast extract. Enterococci and Streptococci were grown in 10 ml TSB plus yeast without agitation under 5% CO_2_. Overnight cultures were harvested at 2,560 g for 10 min. After discarding the supernatant the pellet was washed in 1 ml TE (10 mM Tris-HCl, pH 7.5 and 1 mM EDTA) and recovered by centrifugation at 17,900 g for 10 min. Cell pellets were used for DNA preparation. Clinical samples were obtained from the routine microbiological laboratory were they were characterized by subculture and standard biochemical identification (VITEK2).

### DNA template preparation

Total bacterial DNAs were extracted and purified by using the Bacterial Genomic DNA Purification Kit (EdgeBioSystems, Gaithersburg, MD, USA) following the instructions of the supplier. For Gram-positive bacteria the cell pellets were resuspended in 200 μL TES buffer (20 mM Tris-HCl, pH 7.5, 10 mM EDTA pH 8.0 and 50 mM NaCl) containing lysozyme (Sigma, Taufkirchen, Germany) in a final concentration of 0.8 g/L prior to extraction. In additon, lysostaphin (Sigma) was added to a final concentration of 0.2 g/L, to promote Staphylococcal lysis, or mutanolysin (0.5 U/μL; Sigma) was added to lyse Streptococci and Enterococci and incubated one hour at 37°C. *Candida albicans *DNA extraction was achieved by beating the cell pellet with glass beads (425–600 microns, Sigma) using a Tissue Lyser (Qiagen, Hilden, Germany) at maximum speed for 5 minutes and the DNeasy Tissue Kit (Qiagen) with an overnight Proteinase K (10 mg/L) treatment. DNA from cotton swabs was prepared by DNeasy Tissue Kit (Qiagen) followed by manufacturer's protocol for the purification of genomic DNA from Gram+ bacteria.

### Construction of the prototype microarray

A total of 930 gene segments of *Staphylococcus *spp., *Streptococcus *spp., *Enterococcus *spp., *Proteus *spp., *Klebsiella *spp., *Stenotrophomonas *sp., *Enterobacter *sp., *Acinetobacter *spp., *E. coli*, *P. aeruginosa*, and *Candida albicans *and genes encoding resistance against antimicrobials were selected from the literature and databases. Next they were compared by BLAST analysis to all other sequences available in the NCBI database in order to avoid regions homologous with genes of other bacterial species and *Homo sapiens*. Primers for the selected sequences were designed with the help of Primer3 search [19] in order to produce amplicons of 200 to 800 bp length (primer sequences and their characteristics are shown in Additional file 1).

Negative controls comprising genes of *Homo sapiens*, *Dictyostelium discoideum*, *Mus musculus *and *Hordeum vulgaris *and positive controls (16S rRNA genes of several bacterial species) were also included. PCR products were cloned following the detailed protocol described elsewhere [2]. All cloned gene segments were amplified from the plasmids and diluted in 25% DMSO at a concentration of 200 mg/L. For printing the microarrays a BioRobotics Microgrid 610 spotter (Genomic Solutions, Huntingdon, UK) and Ultra-GAPS™ coated glass slides (Corning Incorporated, Corning, USA) were used and conditions for printing were as described [20]. The complete array of 930 gene amplicons was spotted in 2 replicates per slide, each replicate containing 2 spots of the same probe, therefore totaling 4 replicates of each probe. Each lot of microarrays was quality controlled by hybridization with 2 μg genomic DNA of reference strains of pathogens present on the array.

### Multiplex PCR

For testing the Large-scale Multiplex PCR (LSplex) approach, 800 primer-pairs were selected out of the 930 available primer-pairs. (Additional file 1).

LSplex was carried out with different amounts of pure culture bacterial DNA templates. A primer mix was used with a final concentration of in general 0.02 μM of each primer. Reactions in a total volume of 50 μL were performed with 2 U either of Taq DNA polymerase (Fermentas, St. Leon-Rot, Germany) (*standard LSplex*) or Vent exo^- ^DNA polymerase (New England Biolabs, Frankfurt am Main, Germany) (*optimized LSplex*). Standard LSplex using Taq DNA polymerase amplification reactions contained 1× KCl PCR buffer (Fermentas), 2 mM MgCl_2_, and 0.2 mM of dATP, dCTP, gGTP, and dTTP (Sigma). Optimized LSplex using Vent exo^- ^DNA polymerase amplification reactions contained 1× ThermoPolBuffer (New England Biolabs), 4 mM MgCl_2_, and 0.2 mM of dATP, dCTP, dGTP, and dTTP (Sigma). The cycling was performed in Trio T3 Thermocycler (Biometra, Goettingen, Germany) using protocol comprising an initial denaturing step at 94°C for 3 minutes, followed by 35 cycles of 94°C for 30 s, 55°C for 45 s and 72°C for 1 min. LSplex products were spin purified with the QIAquick PCR Purification Kit (Qiagen) and eluted with nuclease-free water (pH 8).

### Labelling of multiplex amplified products for microarray hybridization experiments

LSplex amplified products were labelled with fluorophores after or during amplification.

#### 1. Labelling after amplification

Purified LSplex products in a volume of 20 μL were labelled with 3 μL of either Cy5-dCTP or Cy3-dCTP (Amersham Pharmacia Biotech Europe, Freiburg, Germany) by random priming using Klenow Polymerase (50 units) (BioPrime DNA labelling Kit, Invitrogen, Karlsruhe, Germany) in the presence of 0.12 mM dATP, dGTP and dTTP and 0.06 mM dCTP, in a total volume of 50 μL. After 2 hours incubation at 37°C, the reaction was stopped by adding 5 μL of 0.5 M EDTA.

#### 2. Labelling during amplification

Labelling during PCR was performed directly, by incorporation of fluorescent nucleotides, or indirectly by incorporation of aminoallyl-modified nucleotides and subsequent staining of the amplified products with amino reactive fluorescent dyes. The LSplex PCR protocols using Taq or Vent exo^- ^DNA polymerases were modified as follows: 1) for direct labelling the amount of dTTP was reduced to 0.15 mM and 0.05 mM of Alexa Fluor 546-14-dUTP was added (ChromaTide Labelled Nucleotides, Molecular Probes, Willow Creek, US). 2) for indirect labelling the amount of dTTP was reduced to 0.13 mM and 0.07 mM aminoallyl-dUTP was added (ARES DNA labelling Kit, Invitrogen). Amino-modified amplified DNA was spin purified with the QIAquick PCR Purification Kit (Qiagen), eluted in 60 μL nuclease-free water (pH 8), analyzed by spectrophotometry, freeze-dried (Lyovac GT2, Finn-Aqua, Huerth, Germany), resuspended in 5 μL nuclease-free water and subsequently stained with Alexa-fluor 555 or 647.

#### 3. Labelling of genomic DNA

For some comparative experiments, bacterial or fungal pure culture genomic DNAs have been fragmented by sonification (Bandelin, Berlin, Germany) to an average size of 1000 bp and then also been labelled by random priming and Klenow Polymerase as described above (1. Labelling after amplification).

Finally, labelled LSplex products and genomic DNA were spin purified with the QIAquick PCR Purification Kit (Qiagen) and eluted in 60 μL elution buffer (10 mM Tris/HCl, pH 8.0). The labelling efficiency was evaluated by calculating the approximate ratio of bases to dye molecules. This ratio and the amount of recovered labelled DNA was determined by measuring the absorbance of the undiluted purified LS-Plex products at 260 nm and the absorbance of the dye at its absorbance maximum using a lambda40 UV-spectrophotometer (PerkinElmer) and plastic disposable cuvettes for the range from 220 nm to 700 nm (UVette; Eppendorf, Hamburg, Germany).

### Microarray hybridization and analysis

In order to provide a complete evaluation of the LSplex protocol using genus-specific and high complexity primer mixes, amplified products were hybridized to a prototype microarray designed to identify pathogenic microorganisms involved in sepsis.

All amplifications were performed at least twice for each condition indicated. Each experiment described in the present study represent co-hybridization of two different DNA samples (LSplex amplified and genomic DNA for comparison) labelled with Cy3, Alexa 546 or Alexa 555 and Cy5 or Alexa 647 respectively. After purification, DNA samples labelled with distinguishable fluorophores were pooled and 10 μg of Salmon Sperm DNA were added. The whole yield of one amplification reaction was used for one labeling and hybridization experiment. The mixture was frozen in liquid nitrogen and freeze-dried (Lyovac GT2, Finn-Aqua, Huerth, Germany) in the dark. Hybridization was automatically performed with a TECAN hybridization station (HS400, TECAN, Salzburg, Austria). The microarray slides were prewashed with 5 × SSC then 110 μL of pre-hybridization buffer (25% Formamide, 5 × SSC, 0.1% SDS, 10 mg/ml BSA) were added and incubated for 30 minutes at 42°C with mild agitation. Lyophilized labelled DNA was resuspended in 110 μL of hybridization buffer (25% Formamide, 5 × SSC, 0.1% SDS), denatured for 3 minutes at 90°C, and injected into the hybridization chambers. Hybridization was performed for 18 hours at 42°C. After hybridization the arrays were automatically washed at 42°C in 1 × SSC/0.1% SDS, three cycles of 30 sec wash time and 2 min soak time, then in 0.1 × SSC/0.1% SDS, five cycles of 30 sec wash time and 2 min soak time, in 0.1 × SSC, four cycles of 30 sec wash time and 2 min soak time and finally dried at 30°C with N_2 _(270 MPa) for 5 min.

Hybridized arrays were scanned with a GenePix Personal Axon 4100A laser scanner (Axon Instruments, Union city, CA). Laser light of wavelengths at 532 and 635 nm were used to excite Cy3/Alexa546/Alexa555 dyes and Cy5/Alexa647 dyes, respectively. Fluorescent images were analyzed by the GenePixPro software (v.6.0) and Acuity (v.4.0) (Axon Instruments). The intensity of fluorescence of each spot was measured and the mean of 4 replicate spots per probe was calculated. Local background fluorescence was also measured and subtracted from the mean fluorescence. Spots displaying fluorescence greater than mean fluorescence of all spots on the array plus two times standard deviation (SD) were considered as positive. The hybridization was considered successful if spiked and control spots produced positive signals. Presence of more than 5 positive spots from same species was interpreted as positivity of the sample for this pathogen species. The fidelity limit of LSplex was defined as minimal amount of DNA necessary to obtain the hybridization pattern with >95% correspondence to one from the 2 μg genomic DNA.

## Results

We have recently established a prototype medium-density gene-segment DNA microarray for the detection and genetic profiling of pathogens causing bloodstream infections [2]. The limit of detection of such medium-density gene-segment DNA microarrays was previously identified and ranged between 10 and 100 ng of DNA [2]. This microarray has been extended for the present study to represent specific gene fragments of more than 20 of the most prominent causative agents of sepsis [15]. As expected the sensitivity of detection was not influenced by the extension of the microarray. This was confirmed experimentally by hybridizing decreasing amounts of bacterial genomic DNA (Additional file 2). At the nanogram level a striking reduction in the detection power was observed and the number of detected genes was gradually reduced. In order to improve the sensitivity of detection we focused on the development of an amplification protocol by multiplex PCR.

### Large scale multiplex PCR with 800-primer pairs (LSplex)

**Figure 1:**
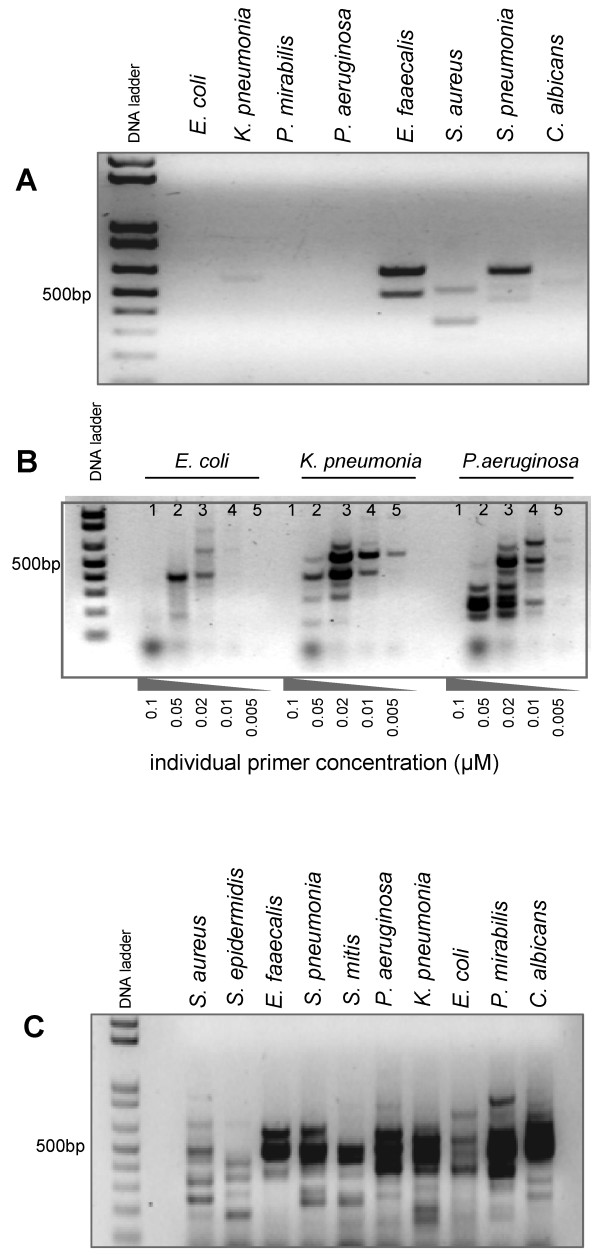
**Large scale multiplex PCR with 800 primer pairs**. Gel electrophoresis of PCR products obtained with high complexity 800-primer pair mix (Additional file 1) with a final concentration of 0.02 μM for each individual primer pair and using Taq polymerase (standard LSplex) (A) or using vent exo-polymerase (B and C). Efficiency of LSplex using primer mix with different individual primer concentrations (B). Optimized LSplex amplification of various DNA templates from Gram-negative, Gram-positive bacteria and *Candida albicans *(C). 100 ng genomic DNA from each indicated species served as template.

The amplification of unidentified pathogen DNA requires that all necessary primer pairs are present in the amplification mix. We have initially addressed the question whether it is possible to amplify genomic DNA of several bacterial species by a PCR containing 800 primer pairs (Additional file 1). However, the complexity of the primer mix did not allow the amplification of any genomic DNA at a final primer concentration of 0.2 μM (data not shown). Nevertheless, reducing the primer concentration in the amplification reaction to 0.02 μM permitted amplification from 100 ng of some DNA templates, although the amplification of most DNA templates was very weak (Fig 1A). It was not possible to further decrease the final concentration of individual primers without a negative effect on the amplification yield (not shown). Furthermore, DNA templates from Gram-negative bacteria could not be amplified using Taq DNA polymerase at any primer concentration (not shown). An optimized protocol using Vent exo^- ^DNA polymerase permitted the amplification of these templates with 800-primer pairs at different concentrations (Fig. 1B). LSplex produced patterns corresponding to the expected size range of PCR products, where each band represents the collection of many amplicons of approximately the same size. Furthermore, absence of amplification was observed in reactions without or with unrelated DNA (e.g. human genomic DNA) indicating specific amplification of bacterial DNA (data not shown). Best results were obtained with final primer concentrations between 0.01 and 0.05 μM and with a primer concentration of 0.02 μM we successfully amplified an expanded panel of test species including Gram-positive and Gram-negative bacteria as well as *Candida albicans *DNA (Fig. 1C).

### Adapting LSplex to microarray hybridization

To demonstrate specificity of LSplex the amplified DNA was fluorescently labelled and hybridized with the pathogen-specific microarray.

**Figure 2:**
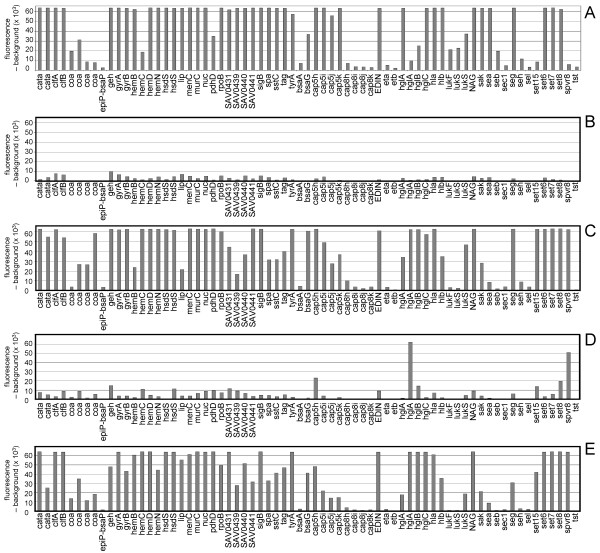
**Microarray detection of LSplex amplification products labelled by different techniques**: Hybridization pattern of specific capture probes obtained upon hybridization of 2 μg (A) and 10 ng of *S. aureus *DNA (B) served as standard for comparison of the profiling fidelity and sensitivity of three labelling protocols for LSplex. LSplex amplification of 10 ng *S. aureus *DNA with subsequent labelling by random priming (C). Direct incorporation of Chromatide Alexa Fluor 546-47-dUTPs during LSplex amplification (D). Indirect labelling by incorporating amino-modified nucleotides during LSplex and subsequent coupling with amino reactive dyes (E).

In microarray analysis the labelling of genomic DNA by random priming and the incorporation of nucleotides tagged with fluorophores is accomplished using the Klenow fragment of the DNA polymerase. This method was employed for LSplex amplified products obtained from 10 ng of *S. aureus *DNA template. The final amount of labelled DNA was high (1.3 μg) and the incorporation of fluorescent nucleotides was efficient (1 nucleotide each 61 bases) (Table 1). The hybridization of Klenow labelled LSplex products reliably reproduced the probe profile obtained with 2 μg of Klenow-labelled genomic DNA (Fig. 2A and 2C). All specific probes that did not hybridize with genomic DNA of *S. aureus *ATCC 29213 were still negative after amplification. For instance those identifying the serotype 8 (cap8 genes), exfoliative toxins A (eta) and B (etb), enterotoxin B (seb), C (sec), H (seh) and L (sel) or toxic shock syndrome toxin-1(tst) (Fig. 2A and 2C).

**Table 1:** Comparison of LSplex labelling methods

Labelling Method	Description	Final amount of DNA^1 ^(μg)	Base/Dye ratio^2^	Labelled nucleotides	Processing time
Random Priming	labelling after amplification with Klenow DNA polymerase	1.3	61	dCTP-Cy3	1.5 h LSplex, 15 min purification; 2 h labelling, 15 min purification
Chromatide	direct incorporation of fluorescent nucleotides during Lsplex	0.7	139	Alexa Fluor 546-14-dUTP(1:3)^3^	1.5 h LSplex, 15 min purification
ARES	incorporation of amino-modified nucleotides during Lsplex staining with Amino-reactive dye	1.1	64	aminoallyl-dUTP (1:2)^4 ^stained after PCR with Alexa Fluor 555	1.5 h Lsplex, 15 min purification; 1 h post staining, 15 min purification

1. Amplified DNA estimated after the last purification step. The starting material for all protocols was 10 ng genomic *S. aureus *DNA (ATCC 29213)

2. BDR calculated following the formula: base:dye = (A_base _× Є_dye_)/(A_dye _× Є_base_); A_base _= A_260 _- (A_dye _× CF_260_) Є_dye _is the extinction coefficient for the fluorescent dye (Cy3: 150000 cm^-1^M^-1^; Alexa555: 150000 cm^-1^M^-1^; Alexa 546: 104000 cm^-1^M^-1^) Є_base _here is the average extinction coefficient for a base in double strand DNA (6600 cm^-1^M^-1^) CF: Correction Factor Cy3: 0.08; Alexa 555: 0.04; Alexa 546: 0.21

3. Ratio recommended by the manufacturer for PCR labelling

4. The manufacturer does not provide a protocol for PCR labelling

### Impact of labeling method on the detection efficiency

In order to reduce the number of steps in the labeling procedure and to shorten the labeling time we attempted to label DNA by incorporation of modified nucleotides concomitantly to the amplification procedure. Additionally, the impact of different labeling methods on general LSplex specificity and sensitivity upon microarray hybridization were evaluated.

The possibility of directly incorporating fluorescent nucleotides during LSplex amplification was examined. Chromatide Alexa Fluor 546-47-dUTPs were used for amplification but resulted in a rather weak incorporation ratio (one fluorescent nucleotide each 139 bases) (Table 1). The corresponding hybridization profile of *S. aureus *specific probes was barely more informative than the one obtained with 10 ng of non-amplified genomic DNA (Fig. 2D and 2B).

The indirect labeling of LSplex products by incorporating aminoallyl-modified nucleotides during amplification, with subsequent staining by amino reactive fluorescent dyes, was a potential alternative to Klenow labeling with one tagged nucleotide per 64 bases. Some probes displayed reduced fluorescence when compared to the fluorescence levels obtained with LSplex amplification plus Klenow labeling (Fig. 2E). For example the 2^nd ^catalase probe (cata), the 4^th ^coagulase (coa), bsaG, all capsular polysaccharide type 5 related genes (cap5), the gamma hemolysin (hglA), and the enterotoxines G (seg) and T15 (set15) showed weaker signals but were nonetheless identified as positive. Notably, LSplex amplification combined with indirect labeling granted a saving of one hour time compared to LSplex amplification with subsequent Klenow-labeling (Table 1).

### Limits of sensitivity of LSplex

Next we wished to determine the minimum amount of target DNA efficiently supporting the optimized LSplex amplification protocol. Agarose gel electrophoresis was unable to detect the LSplex amplification products from templates containing less than 10 ng of DNA (10^5^–10^6 ^genomic equivalents) from several bacterial species (not shown).

**Figure 3:**
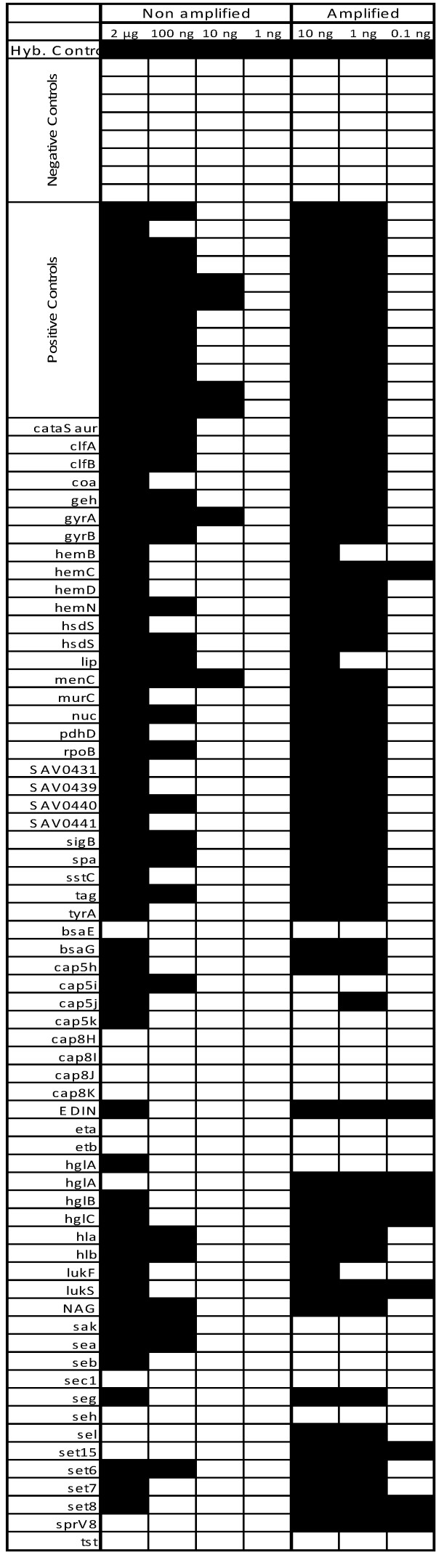
**Enhancement of sensitivity of pathogen DNA detection by microarray by LSplex amplification**. Hybridization profile of non-amplified genomic *S. aureus *DNA (2 μg, 100 ng, 10 ng and 1 ng) and indirectly labelled LSplex amplification product of the same DNA starting from 10 ng, 1 ng and 0.1 ng template (columns). Each row represents individual *S. aureus*-specific capture probes as well as positive (16S-derived probes) and negative controls. Fluorescent signals were quantified and classified as positive (black boxes) hybridization or absence of hybridization (white boxes).

However, after fluorescent labeling of the amplification products followed by microarray hybridization strong signals were readily detected. In fact, LSplex amplification (with 800 primer pairs) of 10 ng and also of 1 ng of DNA template resulted in a hybridization pattern mostly identical to the one obtained with 2 μg of genomic DNA, while 10 ng of the same genomic DNA were below the limit of sensitivity of the microarray for pathogen detection (Fig. 3). The hybridization pattern obtained with 100 ng genomic DNA showed 22 mismatches compared to 2 μg. In contrast, LSplex on 1 ng template displayed a hybridization profile comparable to the one obtained with 2 μg of non amplified DNA, although the amplification of certain probes was diminished. For instance, lipase (lip) delta-aminolevulinic acid dehydratase (hemB) and Pantone-Valentine leukocidin F subunit (lukF) were poorly amplified and fell below detection threshold. Most of the LSplex products amplified from 0.1 ng or 0.01 ng (not shown) template were below the limit of detection of the microarray analysis, making species identification impossible. Thus application of LSplex increases the microarray detection of target templates by a factor of 10^2 ^to 10^3 ^with >95% fidelity.

### Specificity of LSplex on several DNA templates

**Figure 4:**
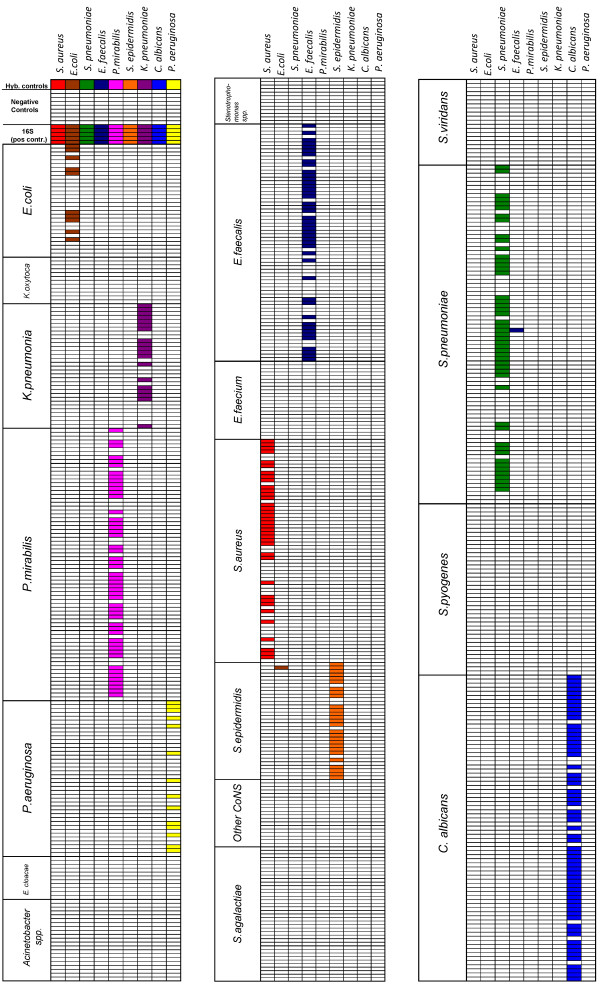
**Specific detection of microbial DNA by LSplex amplification**. Hybridization profiles generated by analysis of LSplex amplified products shown as columns (*S. aureus, E. coli, S. pneumonia, E. faecalis, P. mirabilis, S. epidermidis, K. pneumoniae, C. albicans and P. aeruginosa*). Each row represents an individual capture probe of the microarray, grouped by species or genus specific regions (see Additional file 2) as indicated in the left column. The boxes represent the positive hybridization signal of bacterial DNA (in colour) or absence of hybridisation (in white) with individual capture probes.

In the next step we evaluated if the PCR amplification employing 800 primer pairs results in the generation of nonspecific amplification products cross-hybridizing with non-target species. The specificity of the LSplex protocol was evaluated by measuring the fluorescent signals in the whole array after hybridization with LSplex products (800 primer pairs) obtained from 10 ng of DNA (approx 10^5 ^genomic equivalents) of 9 different species of pathogens (*Staphylococcus aureus *ATCC 29213, *Escherichia coli *ATCC 25922, *Streptococcus pneumoniae *CIP 106577, *Enterococcus faecalis *ATCC 29212, *Proteus mirabilis *DSM 788, *Staphylococcus epidermidis *ATCC 12228, *Klebsiella pneumoniae *DSM 681, *Candida albicans *ATCC 10231 and *Pseudomonas aeruginosa *ATCC 27853). Results are summarized in figure 4. As shown above, LSplex of *S. aureus *DNA allowed unambiguous species identification and discrimination from coagulase negative Staphylococci. Hybridization profiles of LSplex products corresponded very well with the expected hybridization profiles from genomic DNA (not shown). Amplified *S. epidermidis *DNA hybridized specifically to *S. epidermidis *capture probes and showed no cross-hybridizations with *S. aureus *capture probes as well as with capture probes of other coagulase negative staphylococci. Similar results were obtained with LSplex products of *S. pneumonia *DNA leading to clear-cut species identification and differentiation from all other Streptococci species. LSplexed *E. faecalis *DNA displayed high specificity to probes of *E. faecalis*, showing no cross hybridization with the closely related species *E. faecium*. The same was observed in hybridization experiments with *P. mirabilis *DNA. Notably, LSplex products of 10 ng *C. albicans *DNA produced highly specific signals, with 4 to 5-times greater fluorescence intensity than those produced by 2 μg of genomic DNA.

### Application of LSplex for microbiological diagnostics

**Figure 5:**
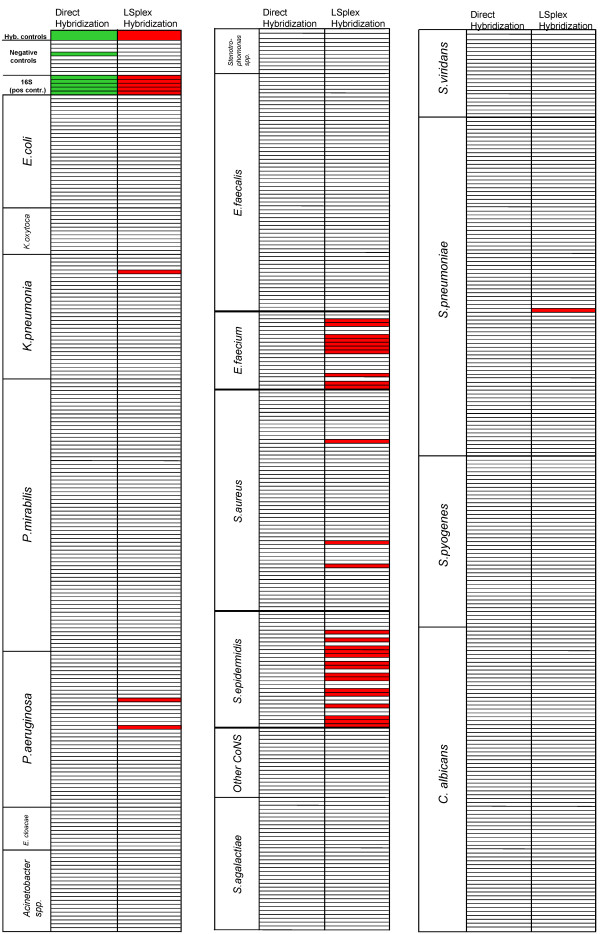
**Application of LSplex for detection of bacterial mixtures from clinical specimens**. Hybridization profiles generated by DNA isolated from cotton swab of superficial wound. DNA was labeled prior to hybridization without amplification (green) or after LSplex (red). Each row represents an individual capture probe of the microarray, grouped by species or genus specific regions (see Additional file 2) as indicated in the left column. The boxes represent the positive hybridization signal of bacterial DNA (in color) or absence of hybridization (in white) with individual capture probes. The presence of *E. faecium *and *S. epidermidis *on swab was verified by routine microbiological diagnostic procedures.

In order to demonstrate benefits of LSplex for the microarray-based detection of pathogens in clinical specimens we analysed cotton swabs taken from patients with superficial wounds. Such swabs represent one of the most frequent materials processed by microbiological diagnostics. Swabs from superficial wounds contain one or more pathogens, normal skin flora and few human cells. The number of bacteria on swabs is usually low, so that time consuming amplification via subculture on microbiological media is required. DNA was isolated from three swabs taken from the same patient. DNA preparations were pooled and divided into two samples of approximately 20 ng each. One sample was subjected to LSplex (800 primer pairs). Other labeled directly prior to hybridization with the microarray. A typical hybridization pattern is depicted in figure 5. The directly labeled DNA hybridized only with 16S RNA probes (positive controls) indicating the presence of bacterial DNA in the sample (Fig. 5). However, the hybridization of directly labeled DNA did not allow identification of the pathogen species. This was not due to inefficient labeling of the DNA as demonstrated by strong hybridization of the control DNA spiked into the labeling reaction. In contrast, LSplex amplified swab DNA hybridized with probes of *Enterococcus faecium *and *Staphylococcus epidermidis *(Fig. 5). The presence of these bacterial species was confirmed by routine microbiological culture followed by biochemical characterization. It should be noted that LSplex of the DNA from swab resulted in hybridization of a few probes from other bacteria (one of from *K. pneumoniae*, two from *P. aeruginosa*, three from *S. aureus *and one from *S. pneumoniae*) which were not identified by microbiological culture. These, however were only singletons in the redundant set of dozens of species-specific probes, allowing the correct identification of pathogens present in the specimen. In summary the results of LSplex amplification of DNA from cotton swabs followed by microarray were in concordance with the standard microbiological techniques, whilst direct microarray identification of the pathogens was not successful.

## Discussion and conclusion

The applicability of fluorescence-based DNA microarrays for the direct detection and characterization of pathogens depends on amplification of the target DNA [21]. To compensate for the low sensitivity of such a multi-capture probe detection system, microarray analysis can be preceded of pathogen isolation and clonal expansion as a source for abundant DNA. A pre-amplification of the target DNA using a single-step Large Scale multiplex PCR (LSplex) could avoid such a time-consuming procedure. Although it is generally accepted that Multiplex PCR is potentially an ideal co-adjuvant for DNA microarrays in pathogen detection [21] there is, nevertheless, a limitation in the number of distinct PCR products that can be generated. Up to date, multiplex PCR was only combined with low-density microarray formats [22] and required either several parallel multiplex PCR reactions [5,17,23] or subsequent PCR steps [6,24].

The complex nature of the interference between multiple primer pairs and targets [25,26,21] has limited conventional multiplex PCR in solution phase to a dozen of primer pairs [27-29]. Antagonizing the typical hindrances of highly multiplexed PCR requires innovative technical platforms as for instance performing on-chip amplification with primers attached to a solid support [26]. Another alternative approach applied to solution-phase highly multiplex PCR has been the replacement of target-specific primers with universal ones. However, this process involves multiple steps starting with enzymatic digestion of the template DNA, ligation to adapters, primer extension and finally two subsequent PCR reactions [30,31]. Such multi-step approaches are time consuming and prone to contamination [25] and therefore have not been recommended for bacteriological routine diagnostics.

The coupling of a pre-processing multiplex PCR to a medium-density microarray format, displaying hundreds of probes for identification and virulence profile typing of several pathogenic species, requires an unbiased multi-target amplification corresponding to several dozens of specific capture probes characterizing a certain pathogen. Since the presence and concentration of the particular pathogen in a microbiological laboratory is unknown, the multiplex reaction should include as many primer pairs as capture probes are present on the microarray. Moreover, the reaction has to cope with femtograms of pathogen template DNA whose GC-content can range between 30 and 70% and which is mixed with nanograms of human DNA.

We have shown high fidelity amplification of specific DNA targets using pools of species-specific mixes of up to 800 primer pairs, which improves the sensitivity of the microarray detection of pathogens by a factor of 2 to 3-logs.

By using *S. aureus *DNA (strain ATCC 29213) as template for amplification, we demonstrated that LSplex tolerates the increase in primer mix complexity until at least 800 primer pairs, without significant reduction in the profiling fidelity. LSplex products amplified from 10 and even 1 ng of template generated fluorescent signals as strong as those produced by micrograms of genomic DNA. Nevertheless, the comparison between LSplex hybridization profiles and the ones obtained with 2 μg of *S. aureus *showed that some probes were poorly amplified with the high complexity primer mixes. These probes produced a strong fluorescent signal when hybridized with genomic DNA but upon the LSplex protocol they were not considered as positive since their fluorescence difference was less then 2 times SD to the mean fluorescence intensity of the whole microarray. This problem of under-amplification of some targets might be circumvented by a specific increase in the concentration of primer pairs amplifying these specific targets [32]. Such a balancing strategy for individual primer pairs could be applied on the whole set of primers, following a broad comparison between hybridization profiles generated by genomic DNA of many reference strains of all species of interest and the LSplex amplified products. In this way, the amount of all primer pairs responsible for low amplification yield can be adjusted. Cut-off values supporting the decision between positive or negative signals are determined empirically and should be specifically adapted to different experimental setups. Although several calculation methods are described in the literature, they basically represent subjective evaluation of the signal to noise ratio. Some authors consider a signal positive when it is only two or three times higher than the assay background [33,16], while others take only signals ten times higher [23].

The fact that the LSplex protocol could allow concomitant amplification and labelling represents a valuable feature for future application in diagnostics since it should reduce the total time required for providing the identification of the pathogen. The optimized LSplex protocol using Vent exo^- ^performed reliable amplification and efficient incorporation of amino-allyl modified nucleotides, allowing indirect labelling of PCR products. However, direct incorporation of fluorescent nucleotides during the multiplex PCR under the same amplification conditions led to weak label incorporation making the separate labelling step necessary to achieve a good profiling fidelity. Alternatively, the use of labelled primers can be employed for obtaining fluorescent multiplex PCR products [34].

LSplex successfully amplified less than 10 nanograms of DNA from several different pathogens (Gram-positive, Gram-negative and fungi) generating signals in general stronger and more specific than the ones generated with 2–5 micrograms of DNA. LSplex improved the specificity of the hybridization assay and enriched the sample for the target sequences present in the template. Interestingly, *Candida albicans *produced non-detectable signals when 2 μg of genomic DNA are used for hybridization. After amplification of 10 ng of *C. albicans *DNA by LSplex protocol resulted in the clear hybridization pattern (Fig. 4).

We would like to emphasize that a reduction in the limit of sensitivity of the LSplex protocol to picograms or to femtograms would be desirable in order to detected pathogens directly from every clinical, food or environmental samples.

In the last two years the publication of several reports referring to rapid identification of bacterial species by multiplex PCR coupled to microarrays detection [5,35,17,16-38,17,3,37,3,4,23,7] demonstrated the usefulness of this approach and the growing interest in implementing it in routine diagnostics. It also underlines the necessity of finding robust protocols for amplifying the target DNA before microarray analysis.

Whole genome amplification (WGA) is a powerful technique for the amplification of total genomic DNA (e.g. for comparative hybridization [39]). However, the random priming employed in WGA will amplify every DNA in the sample. Therefore, the application of WGA is difficult if the DNA of interest is contaminated by unwanted DNA. This is the case in clinical microbiology settings where DNA extracted directly from patient sample contains a significant amount of human DNA. LSplex would amplify selectively the underrepresented bacterial DNA. The large set of primer pairs is potentially able to amplify as many gene segments as probes are immobilized on the prototype microarray but in practice, it is supposed to only amplify the gene-segments specific to the pathogens present in the analyte.

In parallel, real-time PCR-based assays for identification of pathogens were proposed since the sensitivity is adequate for direct detection and quantification [10-12,40-43]. However, the information level obtained by this approach is incomparably lower than the one provided by medium or high density microarray analyses. Real-time PCR has a reduced potential for multiplexing because the current availability of only four to five channels for the simultaneous non-overlapping detection of different fluorophores [21]. For this reason, real-time PCR is in general confined to a mere species identification based on single sequence polymorphism [10,43] or to confirm the presence of a suspected pathogen by using a reduced number of specific primer pairs [44,45] eventually completed by the detection of a few genes related to antibiotic resistance [46,45]. In contrast, microarrays offer the possibility to profile pathogens by providing information at the strain level [36], by detecting virulence factors and genes determining the antibiotic resistance [16]. The LSplex amplification protocol is a promising co-adjuvant for pathogen profiling by microarray analysis since it increases sensitivity and the specificity of detection. It also presents the flexibility of using hundreds of primer pairs, whose sequences are exchangeable in function of the pathogens targeted in the microarray. The single-step LSplex protocol, allowing labelling during amplification, could represent one piece of the methodological mosaic in a future time-saving bacteriological diagnostic approach.

## Authors' contributions

MPS established and performed LSplex PCRs, BEC performed microarray hybridizations, LE designed and produces microarrays, MK and OK performed data analysis and wrote manuscript. All authors contribute to the final manuscript and approved it.

## Supplementary Material

Additional file 1 **Microarray probes and primer sequences.** The table contains the description of microarray probes and primer sequences used in the study. Click here for file

Additional file 2 **Prototype DNA microarray for detection of common pathogens.** The figure represents the analysis of microarray hybridizations with decreasing amounts of bacterial DNA. Click here for file

## References

[B1] Cho JC Tiedje JM Quantitative detection of microbial genes by using DNA microarrays Appl Environ Microbiol 2002 68 1425 1430 11872496 10.1128/AEM.68.3.1425-1430.2002 PMC123775

[B2] Cleven BE Palka-Santini M Gielen J Meembor S Krönke M Krut O Identification and characterization of bacterial pathogens causing bloodstream infections by DNA microarray J Clin Microbiol 2006 44 2389 2397 16825354 10.1128/JCM.02291-05 PMC1489523

[B3] Foldes-Papp Z Egerer R Birch-Hirschfeld E Striebel HM Demel U Tilz GP Wutzler P Detection of multiple human herpes viruses by DNA microarray technology Mol Diagn 2004 8 1 9 15230636 10.1007/BF03260041

[B4] Nordstrom H Falk KI Lindegren G Mouzavi-Jazi M Walden A Elgh F Nilsson P Lundkvist A DNA microarray technique for detection and identification of seven flaviviruses pathogenic for man J Med Virol 2005 77 528 540 16254977 10.1002/jmv.20489

[B5] Panicker G Call DR Krug MJ Bej AK Detection of pathogenic Vibrio spp. in shellfish by using multiplex PCR and DNA microarrays Appl Environ Microbiol 2004 70 7436 7444 15574946 10.1128/AEM.70.12.7436-7444.2004 PMC535186

[B6] Tomioka K Peredelchuk M Zhu X Arena R Volokhov D Selvapandiyan A Stabler K Mellquist-Riemenschneider J Chizhikov V Kaplan G Nakhasi H Duncan R A multiplex polymerase chain reaction microarray assay to detect bioterror pathogens in blood J Mol Diagn 2005 7 486 494 16237218 10.1016/S1525-1578(10)60579-X PMC1888491

[B7] Wilson WJ Strout CL DeSantis TZ Stilwell JL Carrano AV Andersen GL Sequence-specific identification of 18 pathogenic microorganisms using microarray technology Mol Cell Probes 2002 16 119 127 12030762 10.1006/mcpr.2001.0397

[B8] Azara A Piana A Sotgiu G Dettori M Deriu MG Masia MD Are BM Muresu E Prevalence study of Legionella spp. contamination in ferries and cruise ships BMC Public Health 2006 6 100 16620388 10.1186/1471-2458-6-100 PMC1459133

[B9] La Scolea LJ Jr Dryja D Quantitation of bacteria in cerebrospinal fluid and blood of children with meningitis and its diagnostic significance J Clin Microbiol 1984 19 187 190 6365957 10.1128/jcm.19.2.187-190.1984 PMC271014

[B10] Loeffler J Henke N Hebart H Schmidt D Hagmeyer L Schumacher U Einsele H Quantification of fungal DNA by using fluorescence resonance energy transfer and the light cycler system J Clin Microbiol 2000 38 586 590 10655350 10.1128/jcm.38.2.586-590.2000 PMC86153

[B11] Maaroufi Y Heymans C De Bruyne JM Duchateau V Rodriguez-Villalobos H Aoun M Crokaert F Rapid detection of Candida albicans in clinical blood samples by using a TaqMan-based PCR assay J Clin Microbiol 2003 41 3293 3298 12843077 10.1128/JCM.41.7.3293-3298.2003 PMC165319

[B12] Pryce TM Kay ID Palladino S Heath CH Real-time automated polymerase chain reaction (PCR) to detect Candida albicans and Aspergillus fumigatus DNA in whole blood from high-risk patients Diagn Microbiol Infect Dis 2003 47 487 496 14596967 10.1016/s0732-8893(03)00139-1

[B13] Turner NJ Whyte R Hudson JA Kaltovei SL Presence and growth of Bacillus cereus in dehydrated potato flakes and hot-held, ready-to-eat potato products purchased in New Zealand J Food Prot 2006 69 1173 1177 16715823 10.4315/0362-028x-69.5.1173

[B14] Weinstein MP Current blood culture methods and systems: clinical concepts, technology, and interpretation of results Clin Infect Dis 1996 23 40 46 8816127 10.1093/clinids/23.1.40

[B15] Krut O Palka-Santini M Cleven BE Krönke M Analytical device for rapid identification of pathogens 2006

[B16] Vora GJ Meador CE Stenger DA Andreadis JD Nucleic acid amplification strategies for DNA microarray-based pathogen detection Appl Environ Microbiol 2004 70 3047 3054 15128566 10.1128/AEM.70.5.3047-3054.2004 PMC404398

[B17] Xu X Li Y Zhao H Wen SY Wang SQ Huang J Huang KL Luo YB Rapid and reliable detection and identification of GM events using multiplex PCR coupled with oligonucleotide microarray J Agric Food Chem 2005 53 3789 3794 15884798 10.1021/jf048368t

[B18] Odenthal M Koenig S Farbrother P Drebber U Bury Y Dienes HP Eichinger L Detection of opportunistic infections by low-density microarrays: a diagnostic approach for granulomatous lymphadenitis Diagn Mol Pathol 2007 16 18 26 17471154 10.1097/PDM.0b013e31802d6916

[B19] Rozen S Skaletsky H Primer3 on the WWW for general users and for biologist programmers Methods Mol Biol 2000 132 365 386 10547847 10.1385/1-59259-192-2:365

[B20] Farbrother P Wagner C Na J Tunggal B Morio T Urushihara H Tanaka Y Schleicher M Steinert M Eichinger L Dictyostelium transcriptional host cell response upon infection with Legionella Cell Microbiol 2006 8 438 456 16469056 10.1111/j.1462-5822.2005.00633.x

[B21] Petrik J Diagnostic applications of microarrays Transfus Med 2006 16 233 247 16879151 10.1111/j.1365-3148.2006.00673.x

[B22] Mikhailovich V Gryadunov D Kolchinsky A Makarov AA Zasedatelev A DNA microarrays in the clinic: infectious diseases Bioessays 2008 30 673 682 18536036 10.1002/bies.20781

[B23] Sergeev N Distler M Vargas M Chizhikov V Herold KE Rasooly A Microarray analysis of Bacillus cereus group virulence factors Journal of microbiological methods 2006 65 488 502 16242802 10.1016/j.mimet.2005.09.013

[B24] McIver CJ Jacques CF Chow SS Munro SC Scott GM Roberts JA Craig ME Rawlinson WD Development of multiplex PCRs for detection of common viral pathogens and agents of congenital infections J Clin Microbiol 2005 43 5102 5110 16207970 10.1128/JCM.43.10.5102-5110.2005 PMC1248455

[B25] Elnifro EM Ashshi AM Cooper RJ Klapper PE Multiplex PCR: optimization and application in diagnostic virology Clin Microbiol Rev 2000 13 559 570 11023957 10.1128/cmr.13.4.559-570.2000 PMC88949

[B26] Pemov A Modi H Chandler DP Bavykin S DNA analysis with multiplex microarray-enhanced PCR Nucleic Acids Res 2005 33 e11 15661850 10.1093/nar/gnh184 PMC548369

[B27] Kong F Ma L Gilbert GL Simultaneous detection and serotype identification of Streptococcus agalactiae using multiplex PCR and reverse line blot hybridization J Med Microbiol 2005 54 1133 1138 16278425 10.1099/jmm.0.46244-0

[B28] Yang IC Shih DY Huang TP Huang YP Wang JY Pan TM Establishment of a novel multiplex PCR assay and detection of toxigenic strains of the species in the Bacillus cereus group J Food Prot 2005 68 2123 2130 16245717 10.4315/0362-028x-68.10.2123

[B29] Zeng X Kong F Wang H Darbar A Gilbert GL Simultaneous detection of nine antibiotic resistance-related genes in Streptococcus agalactiae using multiplex PCR and reverse line blot hybridization assay Antimicrob Agents Chemother 2006 50 204 209 16377687 10.1128/AAC.50.1.204-209.2006 PMC1346803

[B30] Shapero MH Zhang J Loraine A Liu W Di X Liu G Jones KW MARA: a novel approach for highly multiplexed locus-specific SNP genotyping using high-density DNA oligonucleotide arrays Nucleic Acids Res 2004 32 e181 15601992 10.1093/nar/gnh178 PMC545476

[B31] Broude NE Driscoll K Cantor CR High-level multiplex DNA amplification Antisense Nucleic Acid Drug Dev 2001 11 327 332 11763349 10.1089/108729001753231704

[B32] Henegariu O Heerema NA Dlouhy SR Vance GH Vogt PH Multiplex PCR: critical parameters and step-by-step protocol Biotechniques 1997 23 504 511 9298224 10.2144/97233rr01

[B33] Szemes M Bonants P de Weerdt M Baner J Landegren U Schoen CD Diagnostic application of padlock probes – multiplex detection of plant pathogens using universal microarrays Nucleic Acids Res 2005 33 e70 15860767 10.1093/nar/gni069 PMC1087788

[B34] Lawrence ER Griffiths DB Martin SA George RC Hall LM Evaluation of semiautomated multiplex PCR assay for determination of Streptococcus pneumoniae serotypes and serogroups J Clin Microbiol 2003 41 601 607 12574253 10.1128/JCM.41.2.601-607.2003 PMC149661

[B35] Shang S Chen G Wu Y Du L Zhao Z Rapid diagnosis of bacterial sepsis with PCR amplification and microarray hybridization in 16S rRNA gene Pediatr Res 2005 58 143 148 15985688 10.1203/01.PDR.0000169580.64191.8B

[B36] Call DR Borucki MK Loge FJ Detection of bacterial pathogens in environmental samples using DNA microarrays Journal of microbiological methods 2003 53 235 243 12654494 10.1016/s0167-7012(03)00027-7

[B37] Boriskin YS Rice PS Stabler RA Hinds J Al-Ghusein H Vass K Butcher PD DNA microarrays for virus detection in cases of central nervous system infection J Clin Microbiol 2004 42 5811 5818 15583316 10.1128/JCM.42.12.5811-5818.2004 PMC535236

[B38] Monecke S Ehricht R Rapid genotyping of methicillin-resistant Staphylococcus aureus (MRSA) isolates using miniaturised oligonucleotide arrays Clin Microbiol Infect 2005 11 825 833 16153257 10.1111/j.1469-0691.2005.01243.x

[B39] Silander K Saarela J Whole genome amplification with Phi29 DNA polymerase to enable genetic or genomic analysis of samples of low DNA yield Methods Mol Biol 2008 439 1 18 18370092 10.1007/978-1-59745-188-8_1

[B40] Virolainen A Salo P Jero J Karma P Eskola J Leinonen M Comparison of PCR assay with bacterial culture for detecting Streptococcus pneumoniae in middle ear fluid of children with acute otitis media J Clin Microbiol 1994 32 2667 2670 7852553 10.1128/jcm.32.11.2667-2670.1994 PMC264139

[B41] Wellinghausen N Frost C Marre R Detection of legionellae in hospital water samples by quantitative real-time LightCycler PCR Appl Environ Microbiol 2001 67 3985 3993 11525995 10.1128/AEM.67.9.3985-3993.2001 PMC93119

[B42] Wellinghausen N Wirths B Franz AR Karolyi L Marre R Reischl U Algorithm for the identification of bacterial pathogens in positive blood cultures by real-time LightCycler polymerase chain reaction (PCR) with sequence-specific probes Diagn Microbiol Infect Dis 2004 48 229 241 15062914 10.1016/j.diagmicrobio.2003.11.005

[B43] Jordan JA Durso MB Real-time polymerase chain reaction for detecting bacterial DNA directly from blood of neonates being evaluated for sepsis J Mol Diagn 2005 7 575 581 16258155 10.1016/S1525-1578(10)60590-9 PMC1867550

[B44] van Haeften R Palladino S Kay I Keil T Heath C Waterer GW A quantitative LightCycler PCR to detect Streptococcus pneumoniae in blood and CSF Diagn Microbiol Infect Dis 2003 47 407 414 14522514 10.1016/s0732-8893(03)00129-9

[B45] Warren DK Liao RS Merz LR Eveland M Dunne WM Jr Detection of methicillin-resistant Staphylococcus aureus directly from nasal swab specimens by a real-time PCR assay J Clin Microbiol 2004 42 5578 5581 15583284 10.1128/JCM.42.12.5578-5581.2004 PMC535250

[B46] Palladino S Kay ID Flexman JP Boehm I Costa AM Lambert EJ Christiansen KJ Rapid detection of vanA and vanB genes directly from clinical specimens and enrichment broths by real-time multiplex PCR assay J Clin Microbiol 2003 41 2483 2486 12791869 10.1128/JCM.41.6.2483-2486.2003 PMC156537

