# Supplementary material for: Large scale multiplex PCR improves pathogen detection by DNA microarrays
Source: BMC Microbiol. 2009 Jan 3;9:1. doi: 10.1186/1471-2180-9-1 (PMC2631447; doi:10.1186/1471-2180-9-1)
Supplement: Additional file 1 — Microarray probes and primer sequences. The table contains the description of microarray probes and primer sequences used in the study. [file 1471-2180-9-1-S1.pdf]

Suplemental Table 1. Microarray probes and primer sequences

gene	gi	source species	forward	length	position	reverse	length	position	product
agrB	2981293	*S. epidermidis*	CTTAGGGAAAAAGATGGGTAG	21	31	CGTAAAAATAACTTAGATCACATACAG	27	537	507
agrB2Stalugd	5802558	*S. lugdunensis*	TGCGAATTAAACAGTTAGGC	20	2	TTTTAGTTGCCGATGGTG	18	469	468
agrC	2981293	*S. epidermidis*	CCTCGCATATCAGTTTGTG	19	776	GATGATATTAATCTATTTCCGTTTG	25	1287	512
agrC2Stalugd	5802558	*S. lugdunensis*	TTTAATGTTGTTATTTGTTGCTAAA	25	42	AAATAGAGCATGATCAAAGATAAA	24	491	450
agrCStalugd	18251038	*S. lugdunensis*	TATCAATCTTTCAAGCAGTTATG	23	26	AAATAAAATATAGCGATGGTAAATG	25	491	466
alphSE1368	27466918	*S. epidermidis*	ATGAAGCACAACCACCATAC	20	703	GTATTATTTACGGTCTATTTAGCAAG	26	1236	534
ardeSE0106	27466918	*S. epidermidis*	CCACTTTTTCTACTTCTAAAACTTC	25	240	TATTTTCAAAGATGCAAACACA	22	499	260
ardeSE0107	27466918	*S. epidermidis*	TTGATAACAAACAAATTCTGC	21	13	AACGTTTAAATCTTATCTTAACTG	24	434	422
aroiSE0105	27466918	*S. epidermidis*	TAACACTGAACCCCAATGA	19	562	ATCCGGTTTTTATGGTATGA	20	883	322
atlE	2267238	*S. epidermidis*	CCTCAAACAAGCAGAAAAAG	20	144	ACTAAATGGGGTGAATTGC	19	876	733
bsaE	21281729	*S. aureus*	CGGGATTCTCTGCATTATC	19	160	GTCCGCTGGTATTTCTTTG	19	695	536
bsaG	21281729	*S. aureus*	TTAATTGTTCTACCGCTCCA	20	8	CATTTGTACCCATTATTGTAACC	23	644	637
cap5h	1773339	*S. aureus*	AAAGATAATTGGTTTGCTGAA	21	18	TCAAATCGCTTCTTAATCACT	21	524	507
cap5i	1773339	*S. aureus*	CAAGGGTACTTAAACAAATAGAAAC	25	44	CTCGAATTATGAATGAAGGAG	21	682	639
cap5j	1773339	*S. aureus*	ACTTTGTGCAATTATCAGCA	20	15	ATGAAACAGAAAATATGAAAAATAC	25	394	380
cap5k	1773339	*S. aureus*	TTATGGATAGCGTAAAGACAATAA	24	20	TTCACTGGTAATAAAACAAACAA	23	519	500
cap8H	21203164	*S. aureus*	GATTCTTGGCGCTACTAACA	20	12	CCATCGGTTACATTACTTTCTC	22	533	522
cap8I	21203164	*S. aureus*	TAATGTTTCCTTGCCTTATGT	21	44	TGATAACTTCTTCTTTCACCAA	22	698	655
cap8J	21203164	*S. aureus*	GTAAAAACATTTATGAAATCGAAA	24	7	TGTATGTTCCCCCAATTCTA	20	465	459
cap8K	21203164	*S. aureus*	AAGATACGATTTGTTGATTGTG	22	177	GGCCATACCATAAATACCC	19	738	562
cataSaur	7161886	*S. aureus*	TAAATTGTTTAGATTACAATCAGAGG	26	1293	TTCAAAGTTTTCGTATGTTTCA	22	1512	220
cataSaur	7161886	*S. aureus*	CGTGTTTGGGTTAAATTCC	19	628	ATAATGGTGTGTTCCTCCAC	20	1170	543
clfA	397525	*S. aureus*	AAAAGAAAAACACGCAATTC	20	15	CATTCGTCAACTGATTCGTA	20	715	701
clfB	3393010	*S. aureus*	TAGCATAGCAACAAACAGTGA	21	465	GTTTTGACCTGAAGCTGTATC	21	1164	700
coa	216976	*S. aureus*	GCTGGTAAAGCTGAAGAAAC	20	1021	TACTGTGCCATCTTGATTTG	20	1554	534
coa	216976	*S. aureus*	ACAAGAGAAGCAGTAGCAAAC	21	862	TTGTTTCAGTTGCTTGAGG	19	1366	505
coa	216976	*S. aureus*	AACAAAGCAGATGCGATAGT	20	64	GAAATACGATGCGGTTTATC	20	698	635
coa	216976	*S. aureus*	CTAGGCGCATTAGCAGTT	18	22	TAGCCTTCTCTTCCATTTCA	20	553	532
EDIN	152997	*S. aureus*	AAAGATAGTTCTAAGATAAATGGTC	25	217	GGCCATTATTGGTCTGTTG	19	442	226
epiP-bsaP	21204850	*S. aureus*	CTTAGATGTCCCATGCTGAT	20	85	GTCAAACGAGTGCTAATGGT	20	797	713
eta	153005	*S. aureus*	GCTAGTGCATTTGTTATTCAAG	22	61	TTCACTTCTGTGCATTTGG	19	636	576
etb	153011	*S. aureus*	TTTTAGCAGCGTCAATTTTT	20	29	CTGATCCAGAGTTTCCTACCT	21	658	611
fblStalugd	15341502	*S. lugdunensis*	GAAGTGGAGCGTAATTTGTC	20	121	CTGCTTGAACATCATCTGG	19	766	646
folQShaemolyt	1118000	*S. haemolyticus*	ATGAACATGAGCGACATCA	19	1	ATACGTTCTGCAAGATGTTCTA	22	245	245
gad	23451305	*S. epidermidis*	CTAAGCACAAGAAAGGCTCA	20	5	TCATCATCTATGAATTTACTACTATCC	27	290	286
gcaD	16024896	*S. epidermidis*	GCTTAACAACGTAAAACAAGC	21	108	TCTTTCTCTTCAACAATACGC	21	470	363
geh	153019	*S. aureus*	TTCAATAGGCGTGGTGTC	18	45	TTATCTGTCGGTTTCTCTGG	20	782	738
glucSE1191	27466918	*S. epidermidis*	ACGACGAATGATTCATAAGG	20	11	TTCTATCAATGCTGGATTCAC	21	391	381

gene	gi	source species	forward	length	position	reverse	length	position	product
gyrA	296393	*S. aureus*	AGGCTCGTATGATTGAAAAA	20	809	GGTTTTGAGCACGATATGTAG	21	1588	780
gyrB	296393	*S. aureus*	TTGGCACAACTGATAAGACA	20	494	AAAAATCGTTCAAAGTGCTC	20	1115	622
hemB	2589180	*S. aureus*	ATCATCAGCGACAATGAGAG	20	30	TTTTTAACATCTCGAACTATATCTAA	26	785	756
hemC	2589180	*S. aureus*	GTGCCAATTGCAGGATATG	19	727	TAATGTTGTTCATTTAAGCGTTTT	24	926	200
hemD	2589180	*S. aureus*	TTGATAACATTGCTGTGATAGG	22	194	TTCAATTAGTGATTCGAGTGTT	22	645	452
hemN	14349226	*S. aureus*	TCTTCCATTCTCTCAGTCAAA	21	215	AGACCATGTATGTAGGTGGC	20	971	757
hglA	550421	*S. aureus*	AGAAAGAAAGTGATTTCTATGATT	24	19	TGGCCATATCATTCTTTTAAT	21	348	330
hglA	550421	*S. aureus*	TCGTTACACCGAATGGTC	18	611	AGGTGTGATGCTTTTAATTTTTAC	24	960	350
hglB	550421	*S. aureus*	TTTAAGCGTACTATCACACAGAC	23	753	TATTGTTTTCAGTTTCTTTTGTATC	25	973	221
hglC	550421	*S. aureus*	TTGATAGCGATTTATTTGTAGG	22	611	CAATTCTGTCCTTTCACTTTG	21	947	337
hla	46763	*S. aureus*	GTCAGCTCAGTAACAACAACAC	22	16	GTAGCGAAGTCTGGTGAAAA	20	767	752
hlb	619316	*S. aureus*	TGTTATCGACCGTTTTGTATC	21	158	TTTCATCTTTGGGGATATTTT	21	664	507
hld_orf5	2981293	*S. epidermidis*	AGCTCACGGCTTTAACTATG	20	440	TGTAGAACGTGGAAATGTTG	20	1219	780
hsdS	15922990	*S. aureus*	AGGGCAAATGCTTTCAGT	18	729	AAAAGGATTGTTTCTCTTTTTCT	23	1192	464
hsdS	15922990	*S. aureus*	GTGCCAGAGTTGAGATTCC	19	25	ATTTGTCGGTCGAGTTTG	18	557	533
hsp10	535340	*S. epidermidis*	TTAAACCATTAGGAAATCGTG	21	5	AATAGCTAATATATCTTCTTCATTT	25	276	272
icaA	23345084	*S. epidermidis*	TCAAGACACGCTTTCTAGTG	20	186	ATCGAACCCTTTGTTTCC	18	799	614
icaB	23345084	*S. epidermidis*	GTTTTCTTATTACGAACCACATT	23	428	CTGAAGTGTAAACCCATATTGA	22	756	329
icaC	23345084	*S. epidermidis*	GTATTTACGTGCGTTTATTTG	21	27	ATGAATTGATGAAAAGTGTC	20	527	501
icaD	23345084	*S. epidermidis*	GGTCAAGCCCAGACAGAG	18	3	ATGTCACGACCTTTCTTATATTTT	24	302	300
icaR	23345084	*S. epidermidis*	CATTTAACAGTGAATATACTTGGTC	25	18	GAAATATATCGAAAAAGTGTTGAG	24	423	406
lip	393265	*S. aureus*	TGCATCTTCCATTTTAATAGC	21	48	GTCATTGTCCTTTGTTGGTT	20	768	721
lipShaemolyt	6648931	*S. haemolyticus*	CCAAGATGCTAATGTGTCTTC	21	243	TTTGACCTGGAGTTAAATGG	20	877	635
lukF	551668	*S. aureus*	GGTGTCCTATCTCGAAAACA	20	751	TTTTTCCTTAGATTGAGTATCTATT	25	963	213
lukS	551668	*S. aureus*	ATAAAGAAAGGAAATGATTTTATG	24	31	TATCTTTAACAAAATCAAACTGAA	24	250	220
lukS	551668	*S. aureus*	ATCATTAGGTAAAATGTCTGGA	22	630	AATTATGTCCTTTCACTTTAATTTC	25	988	359
menC	1255258	*S. aureus*	TTGACAGCTTTGCATTTTTA	20	7	GGCTTTGTTGCTTTTAATGA	20	422	416
msrw1Stwar	22218025	*S. warneri*	AAAAGATATGACATAATGTTACGAA	25	272	ATAGTATATGTAGCTGGCGGTTG	23	471	200
murC	2642658	*S. aureus*	GTATTATTGCTTGGGGTGAT	20	596	GGATATTTCTTTCGTGCTGT	20	995	400
mvaCShaemolyticus	9937367	*S. haemolyticus*	TTGAATTGGGAACGACAG	18	80	ACTACTATTTGGGCGAACAC	20	642	563
mvaDShaemolyt	9937371	*S. haemolyticus*	AATGACGATGAAACTTCCTTT	21	469	ACCCATACGCTTGAAATCT	19	699	231
mvaK1Shaemolyticus	9937371	*S. haemolyticus*	CATCAATTGTGTGATAATGATAAG	24	577	GCTGTTTGCAATTCTTTAGC	20	854	278
mvaSSepid	9937375	*S. epidermidis*	TTATTCTGCTATATGATATTCACG	24	1	AACATTAGTAGATGGATTCAAGG	23	220	220
mvaSShaemolyticus	9937367	*S. haemolyticus*	ACGTATATCGTCCTGAATATTTTCTAA	27	22	CTACGCTAGTCGAAGGCTAT	20	221	200
NAG	2506026	*S. aureus*	AAGTTGCTCAAATACAAGCTG	21	760	TGATGTTAGCCCAATCTACA	20	1371	612
nitreSE1972	27466918	*S. epidermidis*	ACATTAAGTCAGCATTTGGAG	21	150	TAACTTTAAGACGACTATCCATCA	24	359	210
nitreSE1974	27466918	*S. epidermidis*	ATTAGAGCCAAAGTACTCTCCAC	23	91	TAACAAACCAACCTTTCGATAC	22	290	200
nitreSE1975	27466918	*S. epidermidis*	TTTGATACCTGTAATTTGTTCTTG	24	2182	CACCAATTGATCCAATGTTAT	21	2465	284
nuc	46623	*S. aureus*	TGGCTATCAGTAATGTTTCG	20	74	GAATCAGCGTTGTCTTCG	18	686	613

gene	gi	source species	forward	length	position	reverse	length	position	product
nukMStwar	9049353	*S. warneri*	CATCCAATTTACAGAACCATC	21	45	CGTCATCAGAAGTAGAAGTGAG	22	559	515
oiamtSE1209	27466918	*S. epidermidis*	AGCTTCTCGCACTACTTGAC	20	121	GAATGAGGCAGCTATCAAAC	20	841	721
ORF1Sepid	2267238	*S. epidermidis*	AAAGAAATTAAGTCTCTAGCCAAA	24	401	TTATTGTGCGTAAGGATAATTG	22	865	465
ORF3bSepid	2267238	*S. epidermidis*	AACCAAACGATGCTAGATGA	20	19	AAGAATACTTTACCAAAAGGTGAG	24	380	362
pdhD	48871	*S. aureus*	ATGGTCAAGCTGCTGAAGT	19	1010	ATCATCTTCTTTAAGTGTAATAAGTTT	27	1221	212
proDStwar	22218025	*S. warneri*	TGTATCAACTCCACTTTATTCATATT	26	2	TTTTTCGTATCTTATGGGTCTT	22	321	320
proMStwar	22218025	*S. warneri*	AGCAAGTTCTTTGTTAATTGC	21	21	TTCGTTTGATTGAACTTGAAC	21	291	271
psm_beta1and2	3212078	*S. epidermidis*	ATGTCAAAATTAGCAGAAGCTAT	23	1	TTAGAAACCGAAGATTTTACCT	22	325	325
purR	16024896	*S. epidermidis*	TGACACAATACCTCATGAACC	21	35	TCCTTTCTTATAACCACTACAGG	23	485	451
qacR	22476845	*S. epidermidis*	AGTTTTATAATATTCAGTGCAAAA	24	241	TGAAAGATAAAATACTAGGTGTCG	24	560	320
RNApolsigm	32396031	*S. haemolyticus*	AATCGTCCACTTGTCTTTTG	20	162	AATTTCACTAGGCGTATAATCAG	23	726	565
RNApolsigmSsapro	32396039	*S. saprophyticus*	AAGAGAACCAAACCAATCG	19	49	TTCTTTCGGCTTAAAGTCAT	20	480	432
RNApolsigmSsapro	32396039	*S. saprophyticus*	GAAATAACCGCATTCCAAC	19	8	GATTTGTATATTTAGTGTTTCAGCA	25	507	500
rpoB	677848	*S. aureus*	TGGAAGACATCGTAAACGTA	20	24	TGGATCAAAGAAACGTGAAT	20	786	763
sak	47425	*S. aureus*	TGTTATTATTCTCATTTTCTTCAAT	25	38	ATGCTCTGATAAATCTGGGA	20	438	401
SAV0431	15922990	*S. aureus*	GGCAGTTGTACTCCCACA	18	1050	CTTTCAACACCCCAAGTTC	19	1549	500
SAV0439	15922990	*S. aureus*	TGAATAGAAATACTAGGACCACAA	24	2	TTAAGTTTAATCCATCTGAAAAAT	24	469	468
SAV0440	15922990	*S. aureus*	GGTGTATTAGATAATGAAGGTATGG	25	265	TCTTTATTTTTGGCAGGTTG	20	776	512
SAV0441	15922990	*S. aureus*	AACCAAAAGGCGAGAGTT	18	254	GCTGTCAACATAATAAATGCTTC	23	755	502
sea	153120	*S. aureus*	TTTTATTCATTGCCCTAACG	20	26	TTTTCAGAGTTAATCGTTTTATTATC	26	740	715
seb	152999	*S. aureus*	CGTAGATGTGTTTGGAGCTA	20	324	CTTGAGCAGTCACCTTTTTC	20	556	233
sec1	46566	*S. aureus*	AATTTTTGGCACATGATTTA	20	209	CTTTTATGTCTAGTTCTTGAGCTG	24	568	360
seg	19352023	*S. aureus*	CCACCTGTTGAAGGAAGAG	19	94	TTTAGGAAATAAGTCAAACCAAA	23	594	501
seh	19352025	*S. aureus*	GAAGATTTACACGATAAAAGTGAG	24	1	AAATCATAAATGTCGAATGAGTAA	24	542	542
sel	14349227	*S. aureus*	AGTCTTATCTAACGGCGATG	20	63	AGTTGTAACTGTTTGATGCTTG	22	405	343
set15	15922990	*S. aureus*	GGATATAAATACGGAAATAAAGTTACA	27	175	GAAGTCACAGAAGTAGTGTGTTG	23	446	272
set6	15922990	*S. aureus*	ATGAAATTTAAAGCGATAGCA	21	1	GTATAAACTACTTTGTGGTTCTTCTTT	27	450	450
set7	15922990	*S. aureus*	GTATTGAATATAAAAATGTGACAGG	25	155	GACATTGATAACATCGGACA	20	654	500
set8	15922990	*S. aureus*	GCGCAATTACAGTAACGAC	19	56	TAGATTGCATTGGTTGTGG	19	439	384
sigB	1729791	*S. aureus*	GCGAAAGAGTCGAAATCAG	19	4	AAGGTGAACGCTCTAATTCA	20	403	400
sigrpoStwar	32396027	*S. warneri*	TATTGTCAAAGTCACAACAATTAGA	25	32	ACGTTGTATCGCATTGTAAA	20	531	500
sin	22476845	*S. epidermidis*	TTTAGAGAGACAGCTAGATAATTTGA	26	42	CAATCTTACTAATTGCTTGACCT	23	544	503
slamStalugd	5802563	*S. lugdunensis*	CAAAGGAGTGTGATTTTATGTC	22	273	TGGATTAGCTTTAGGACCAG	20	784	512
slushABCStalugd	1778750	*S. lugdunensis*	TGTCAGGTATCGTAGATGCAA	21	2	GGAATAATCCAATAATAAAGTCTACA	26	501	500
spa	21281729	*S. aureus*	CCGTTACGTTGTTCTTCAGT	20	587	GCAATGGTTTCATTCAAAGT	20	1115	529
spoVG	16024896	*S. epidermidis*	GTGACAGATGTAAGACTTAGAAAAATA	27	1	AGCTTCGTCTGATTCTTCG	19	300	300
sprV8	46686	*S. aureus*	TTTAAAAGTTAGTTCTTTATTCGTTG	26	15	ACTTGAATATAAGTTACGGGTGCAT	25	284	270
sstC	3724154	*S. aureus*	TGATATTGGAAGATATTAGCATAGA	25	44	TGACAATCGCTTTATTCATTT	21	571	528
tag	6434027	*S. aureus*	TTTTGATTTATCTTCTGACGG	21	48	CATTCATTTTATTCCCACCT	20	560	513

gene	gi	source species	forward	length	position	reverse	length	position	product
tnpStwar	31074829	*S. warneri*	TCAGATATAAACAATTTAACAAGGA	25	11	GAAGGTGCCTGATCTGTAAT	20	410	400
tst	18266750	*S. aureus*	TTTTTATCGTAAGCCCTTTG	20	26	CAATAACCACCCGTTTTATC	20	581	556
tyrA	21204263	*S. aureus*	TGGATATAACAATCAAAATCACTC	24	5	TTTTAGAAATGATTCGACAGC	21	365	361
ureSE1861	27466918	*S. epidermidis*	TGAAAGAAGGGATAGTTTTGC	21	2	TTAAACGATTGGGTGATGG	19	321	320
ureSE1863	27466918	*S. epidermidis*	ATGCAAATTATGGAGATGAAG	21	110	GACAGCTTGACCTTTACCAG	20	612	503
ureSE1864	27466918	*S. epidermidis*	TGATTATAGAAGAAATTCAAGGAAA	25	2	TCAATCATGTGAATGTCCTA	20	453	452
ureSE1865	27466918	*S. epidermidis*	TTAACTTATTCAGATGGGATAGC	23	163	GAAATAAAGATTCGAACATGAAC	23	686	524
ureSE1867	27466918	*S. epidermidis*	TCGTATATGGAATTTGTAGCAG	22	313	TGTCGATGATAAATCGTTTG	20	812	500
yabJ	16024896	*S. epidermidis*	ATGAAAATAATCAACTCAGATAAGG	25	1	TATAATTCCTTCACTTTACCTATCAA	26	380	380
ydhK	18148881	*S. hominis*	ATCGTGCTTTCAGTTCATTT	20	3	ATTATCAGCTTGCTCCAATAA	21	502	500
0487Straga	22533503	*S. agalactiae*	AGTAATGGCCAATTGTTGC	19	225	TGACTAATCCTACTTTCGGTGA	22	455	231
0488Straga	22533503	*S. agalactiae*	AATCCCTTTGGCTTGAGA	18	1915	AAAACTGACCCTAATAATCCAC	22	2154	240
0493Straga	22533503	*S. agalactiae*	CTATTATAAAGAACGAGGGCAAA	23	1290	AGTACTATTCATTTTAGCACGACA	24	1689	400
0495Straga	22533503	*S. agalactiae*	CCCTAAATCAAGCCATAAAG	20	45	TGCAATCATACCACACAAAG	20	672	628
0498Straga	22533503	*S. agalactiae*	AGTTTTATCTATTGACGATTTAAGC	25	477	CAACCGTAATCCCTCTATTATATC	24	864	388
0499Straga	22533503	*S. agalactiae*	GGAGCGCGTTTAGTTTACG	19	310	GCCACTACTTGTCCACCATC	20	539	230
0500Straga	22533503	*S. agalactiae*	TGTTCTTAACCATGCTGTTG	20	39	TCATTGATTGACGAATGTGA	20	448	410
0502Straga	22533503	*S. agalactiae*	TAATGAATGAAGGTGTGGAAC	21	230	ATTCTGACTTCTGGCGTACTT	21	469	240
0504Straga	22533503	*S. agalactiae*	AATGGGCATTTCTCTTATTG	20	41	GTATAGAAGATGAAGCAGCATAAA	24	280	240
1760Strpneu	15459462	*S. pneumoniae*	TTGTCTTGACTGGTTGTGTC	20	53	CTACTCCGTGAAGTGAAAGG	20	598	546
573Stprmut	24376948	*S. mutans*	ACGTCCTCTTAACAGTCACAA	21	42	TGCGACTAGGATGGTTTC	18	561	520
580SStprmut	24376948	*S. mutans*	ATCAGGCTGTTATGCGTCT	19	836	TGCCCATCTTTCATTTCA	18	1298	463
581_582SStprmut	24376948	*S. mutans*	AATTGTGAACCAGTTAGAAACC	22	51	TGCCACCTGATAGTGAGC	18	410	360
584SStprmut	24376948	*S. mutans*	GAAGAAACGTGAACGATTAGA	21	3	TTAATTGGCCATCCAAGA	18	465	463
acyPStrpneu	15459462	*S. pneumoniae*	TGCAAAAGGTTAGAATGATTG	21	2	AAGTCAGAGTAGGGAGGAAAG	21	263	262
CAMPfactor	840865	*S. agalactiae*	ACATATGATGTATCTATCTGGAACTC	26	12	CTGTTAAGGCTTCTACACGAC	21	337	326
CAMPfactor	840865	*S. agalactiae*	TATACAAAATCAAAACTTGATAAGGA	26	556	TTTTAATGCTGTTTGAAGTGC	21	765	210
cap1EStrpneu	1944619	*S. pneumoniae*	ACTCTTGGCTTGATTTTGG	19	478	TTGAAGTCCCATCTCTTTTC	20	1050	573
cap1FStrpneu	1944619	*S. pneumoniae*	AAATATTGTTGGTTTACTTCGTG	23	18	TCATAATTTCATCTCCCTTGA	21	552	535
cap1GStrpneu	1944619	*S. pneumoniae*	CTAAAGCATTTGGAGAGATTG	21	95	TGGATTCTATTCGCTACTCC	20	785	691
cap3AStrpneu	1658316	*S. pneumoniae*	TGTAGGAACTGATTTGGATGA	21	477	CCATAACTCCCTTAACAGCA	20	985	509
cap3BStrpneu	1658316	*S. pneumoniae*	TTTCCAGACATAAACCATCC	20	227	GGCATTTCTAATCATCCAAG	20	921	695
celAStrpneu	3211752	*S. pneumoniae*	TCGTCATCTGTACTGGTCTG	20	41	CTCGTCTACTGACTTGAACTTG	22	582	542
celBStrpneu	3211752	*S. pneumoniae*	GTGGAAATCTGCTGGTAAAG	20	147	GCTCATTCATCTCCTCAAAG	20	691	545
cglAStrpneu	3211747	*S. pneumoniae*	GATTATCGGAGAAATTCGTG	20	609	GATAGTCTCCACCAGATGAAA	21	1010	402
cglBStrpneu	3211747	*S. pneumoniae*	TGGACAAGCAGTGTGTGA	18	2	TAGGTCTGCACAAAGATTCC	20	464	463
cglCStrpneu	3211747	*S. pneumoniae*	AAAAATGATGACATTCTTGAAA	22	6	TTAATCATTGACTTTACGATTTG	23	327	322
cglDStrpneu	3211747	*S. pneumoniae*	ATGCTGGAAAGTCTCTTGG	19	19	TTTGTTTCCTTAATGCGTTT	20	398	380
cinA	2398824	*S. pneumoniae*	GGCTGTAGGAGACAATGAAG	20	120	CTTTGTTGACAGACGTAGAGTG	22	666	547

gene	gi	source species	forward	length	position	reverse	length	position	product
cps14EStrpneum	2198539	*S. pneumoniae*	TTGTTAGAGAGACAGAACTTGAAC	24	80	AGATCGACAAACACCTCATC	20	599	520
cps14FStrpneum	2198539	*S. pneumoniae*	AATATTTTATCCATGTTATTATCCTAC	27	135	GTCGGAGCATCAATTCTATC	20	344	210
cps14GStrpneum	2198539	*S. pneumoniae*	TGAGGGATTTATTCAGGATG	20	75	TTAAAAAGGTTACTGATTTCCAC	23	479	405
cps14HStrpneum	2198539	*S. pneumoniae*	TGCTTTAACTCTTTTACCAACC	22	102	TGGACAGAAAATTCCAATAAG	21	723	622
cps19aHStrpneum	3907597	*S. pneumoniae*	AAGGCAATTCCAATACAAAG	20	71	GATGGTGCAAGACCTTAATC	20	757	687
cps19aIStrpneum	3907597	*S. pneumoniae*	TTTCAATGCCTCTCTTGG	18	209	TCCCAGCTATCAAGCCTAC	19	742	534
cps19aKStrpneum	3907597	*S. pneumoniae*	TCCATTAGTCAATGAGTTGAAA	22	57	GTCACATAAACATTCTCTCTACCTT	25	506	450
cps19fGStrpneum	1881538	*S. pneumoniae*	GAAGCATACGACAAACTTCC	20	169	TCAGGTAGCGATCATACTCC	20	733	565
cps23fGStrpneum	3818479	*S. pneumoniae*	GTGATAGTGAACTTGGGATTG	21	73	AACGCCAGTAAAGAGATGAC	20	734	662
cpsA1Strgal	13022158	*S. agalactiae*	AAAGAAACACTCACATACACCTC	23	27	AAACCATAGCTTGACTATCACC	22	559	533
cpsB1Strgal	13022158	*S. agalactiae*	TAAACACGAAGCGGAAAA	18	186	TCAATAAAAAGTTGGTTAGCAC	22	695	510
cpsC1Strgal	13022158	*S. agalactiae*	TAATCACATTTGTTGCCATT	20	77	CATCCAAAATACCCATAATAACT	23	610	534
cpsD1Strgal	13022158	*S. agalactiae*	CGTACAAACATACAGTTTAGTGG	23	73	TCCATAATCGCCGTAAGTAG	20	657	585
cpsE1Strgal	13022158	*S. agalactiae*	TTTGGAGTCGTGGCTATCT	19	143	GCTAAGTGCCTCTACATTAACA	22	669	527
cpsG1Strgal	13022158	*S. agalactiae*	TGATCAAGAAGTGTTCATTCAA	22	87	TTATGACAAAACATATCATTATTTCC	26	446	360
cpsIStragal	5823209	*S. agalactiae*	TGAGGTTTGATGAGAACTTAAAA	23	512	ATCCCTTTAATGATAAGTATTTGAC	25	895	384
cpsJStragal	5823209	*S. agalactiae*	GGGTTGTCAGAAGCTAGAAA	20	220	TTATTCGCTCGTCTAATGCT	20	733	514
cpsKStragal	5823209	*S. agalactiae*	TATTCTTGGGTGGTTCTGTC	20	368	AGTGGACGAATGTGTTATCC	20	891	524
cpsMStragal	5823209	*S. agalactiae*	TACACTCGGGAATTTATTGG	20	39	AATCAAGGGAACAGACACAG	20	651	613
cpsYStragal	5823209	*S. agalactiae*	TCAGGACTGTTTATTTTTATGATT	24	1	CGACGATAATTCCTTAATTGC	21	451	451
cpsYStragal	5823209	*S. agalactiae*	AAAGTTCGTATTGGGTCA	18	564	ATGAGAATTCAACAATTACAATA	23	924	361
cyclStrpyog	5851899	*S. pyogenes*	TAGCTCATATTGTCGCAAAG	20	23	TGATGGACCTCTGTTACCTC	20	533	511
cylBStraga	6007852	*S. agalactiae*	CGTTTCCTTAGGGACTGTT	19	4	TTGCCAATAAAATAAGAATGAG	22	386	383
cylEStraga	6007852	*S. agalactiae*	TTACCTATGGTTGTTTAGATTGG	23	221	CGAACCCTCTTTACTTGTTC	20	755	535
cylFStraga	6007852	*S. agalactiae*	CTGGTGAAGAATGTGAAGAAG	21	74	AAATATCAGTAATGGGTTGTCC	22	592	519
cylHStraga	6007852	*S. agalactiae*	CGAGCATAAACAGCATCTC	19	57	CCAGAAGAATAGGGCTGAC	19	629	573
cylIStraga	6007852	*S. agalactiae*	TCTTATCATCTGCTGCTTCC	20	18	TGACGAGAGAGGACTTGTG	19	627	610
cylJStraga	6007852	*S. agalactiae*	AGCGATATGATGCTTTCTTT	20	194	GATAATGCCTTGGTAATGTCTT	22	799	606
cylKStraga	6007852	*S. agalactiae*	AAAAATAGGCAGTCTACATTGG	22	10	ATTCTTTTAAGTAAACCGCTTC	22	520	511
dexB	1658316	*S. pneumoniae*	AGAGCAAAAACGCTGGTT	18	315	TAGTAATTCCACACAGAAAGCA	22	694	380
dinF	2398824	*S. pneumoniae*	ACGAACAGTGGACCTGATAC	20	12	TAAACCTTGCGAAATAATGG	20	621	610
dltAStrmut	2952523	*S. mutans*	AGATATGATTGCAACAATTGAA	22	21	CGCATGATTGATTTGATAAG	20	432	412
dltBStrmut	2952523	*S. mutans*	GTGAAAAGTCGCATCAAATC	20	173	GCCATAAACATACATGACTCC	21	732	560
dltCppx1Strmut	2952523	*S. mutans*	CAGCTGTTGCAGAAGATTTAG	21	491	CCATTAGCTGCTTGTGATG	19	740	250
dltDStrmut	2952523	*S. mutans*	TTCTCTTAAATCAAACAGGAAGT	23	470	ACCCATTTGGCATTAACC	18	977	508
endAStrpneu	15459462	*S.pneumoniae*	GTTCGGTAATCCAGTTGAAG	20	26	TTAACAGACGCAGTCAAGAG	20	654	629
exoAStrpneu	153626	*S.pneumoniae*	CGTGGAAATTTTAGAAGAACTC	22	168	CAAGGACTGGTTTTTCTTTG	20	490	323
exp72	1208737	*S.pneumoniae*	AAAATTTGGGGGATTCAGT	19	31	TCATAAGCAGCAGTAACCAA	20	236	206
fah_rph_hlo_Strpyog	15425741	*S. pyogenes*	TTAAATACGCTAAAGCCCTCT	21	247	AGGGTGCTTAATTTGACAAG	20	546	300

gene	gi	source species	forward	length	position	reverse	length	position	product
fasCAXStrdysg	21629521	*S. dysgalactiae*	GACATTGAGATTGGTGATGAG	21	178	TTAAGCGTGAGACCATACAG	20	700	523
fnlAStrpneu	33113982	*S pneumoniae*	CCTCTAAGGCTATGATGGAA	20	425	CTATTAAGGGATTGCGTTTG	20	934	510
fnlBStrpneu	33113982	*S pneumoniae*	TGAAAGATGGACGAGATAGG	20	74	GATGGGCTAGGTAAGATTTG	20	662	589
fnlCStrpneu	33113982	*S pneumoniae*	TACATGGATGCTGTTGGAG	19	223	GATCTAGGATGGCAAGAATATAAG	24	767	545
folDStraga	22533503	*S. agalactiae*	TCGAAACCTTTCTGAGGTAA	20	576	GAACAGGTGTTATCATACTAGCAA	24	775	200
gct18Strpneum	13377447	*S. pneumoniae*	CATATGGTACATTTGATTTATTGC	24	17	GTGTCAATATGGTAGTCTTTAACATC	26	266	250
hexB1	4495245	*S. pneumoniae*	CTCCTAGTGCCCTATATCTTTG	22	4	TACGATGATTGGCCTTGA	18	286	283
hftsHstrpneu	5739310	*S. pneumoniae*	CTTGGTGCACAGAGTCCT	18	68	TTTTCGTCATTCATTTTTGA	20	351	284
hlyXStrmut	2952523	*S. mutans*	GTGATATTATCCAAACCATTCTC	23	707	TTAAAGCGGTCAAAAGGTC	19	919	213
hylStragal	22536185	*S. agalactiae*	CTAGGGAATGGTCTGCTTG	19	368	GAGACGGATTGGTTAGAGTG	20	889	522
igaStrmitis	1870144	*S. mitis*	ATTGGACTTGTGTCAGCTTC	20	55	CCTTTGTCTACCTTCATTGG	20	669	615
igaStrpneu	1460093	*S. pneumoniae*	GTGGATGCTCAAGAAACTG	19	118	CTTATGTTCGGCTAATTGTTC	21	840	723
igaStrsanguis	2288954	*S. sanguinis*	GAAGGCAATGAGGAGACTTAC	21	223	CTACTCGGCCAGACAAGAC	19	763	541
immunofrag1Strpneu	33077817	*S. pneumoniae*	AAGTAGGCGATGGTTATGTC	20	1157	TATTTCCTGAATCCTGATGG	20	1783	627
immunofrag2Strpneu	33077816	*S. pneumoniae*	GCATCTCTCGTTATGTCTTTG	21	1238	TTTGGAGCTTTGTATGTGTG	20	2021	784
immunofrag3Strpneu	33077815	*S. pneumoniae*	GACCATTACCACTTTATTCCTTAC	24	931	TTTGCTCTTCTGTCAAGTCC	20	1432	502
int	1877426	*S. pyogenes*	CCGGTTATGTTAATGGAAAG	20	62	TCAAATCATCCCAAGTTAGTG	21	640	579
int315.5	28876381	*S. pyogenes*	GCTATTTGGCCCTGTGTAG	19	131	CAAACGTAGATCAATGCAAG	20	637	507
kdtBStrpneu	15459462	*S. pneumoniae*	TTTCATTTCTTATTTCCTCCA	21	12	GCTTATTCACAGGCTCATTT	20	473	462
lichStrbov	2231061	*S. bovis*	AATTGTTCTCTTATTGGCTTTC	22	24	CCTGGATTTTTAATAGGTTTCA	22	323	300
lipStragal	22536185	*S. agalactiae*	TCCCACTTAACTATGTTGCTC	21	137	CTTGCCTGACTATTTGATGAC	21	722	586
lysAStrpneu	15459462	*S. pneumoniae*	GATTTCTGTATGAGGCAGTTC	21	184	CTTCCTAGCGTCCAATACC	19	691	508
lytA	4495247	*S. pneumoniae*	ATTATCACTGGCGGAAAGA	19	3	GATAAGAGCCGTCTGAATGT	20	467	465
lytRStprmut	24376948	*S. mutans*	GTCTTGGCGCGAGTTTTA	18	167	TTGAAGCCTTTTGAGCAG	18	432	266
lytSStprmut	24376948	*S. mutans*	CATTGGTTCGATCAGTCAG	19	966	GCTTTGGTAGGATATATTAGTGG	23	1365	400
murEStrpyog	13621628	*S. pyogenes*	GCTCACGGTCACTAATAATCTC	22	147	CTGACAACCAGATCACCAC	19	647	501
nanA	1163109	*S. pneumoniae*	AATGGAATGAACGGAAGTG	19	10	TGTTCTCTCTTTTTCCCTAGC	21	461	452
nanBStrpneu	1163109	*S. pneumoniae*	GGTCAACTGTCCATATCTCC	20	115	GACTGATTTCACCAATACTTCC	22	640	526
neuA1Strgal	13022158	*S. agalactiae*	TGAATCTGGAATGTTTGATAAG	22	114	GACCGATGACGTGGATAA	18	658	545
neuB1Strgal	13022158	*S. agalactiae*	TTATTCTTTCGACGGGTATG	20	410	ATCATCTTGCGCTTCTTG	18	966	557
neuC1Strgal	13022158	*S. agalactiae*	GCTGAATACGGAATAATGAAG	21	34	TGGTTAGAGGAGGAATTGATAG	22	565	532
neuD1Strgal	13022158	*S. agalactiae*	AGTCATGCTGATGCGATT	18	34	CGATGAAATATCCAAGGTTT	20	540	507
oppA	1420857	*S. pyogenes*	CCCAGTTCAATTAGATTACCC	21	1296	TTGACTTAGCCTTTGCTTTC	20	1927	632
oppCStrpyog	1420857	*S. pyogenes*	TTTGATGGTGTTGGGTATG	19	298	AAAGAGGTAGGCATTTGTTG	20	831	534
oppD	1420857	*S. pyogenes*	TCGAAGAGATTTTCTATGATCC	22	731	CAGACATTTCCTTCCTCCT	19	1070	340
pcpBStrpneu	1914873	*S. pneumoniae*	GGCTGTTAGTCCAAGTCAAG	20	246	GCTACCTGTAACCATTGCTC	20	897	652
pcpCStrpneu	1914873	*S. pneumoniae*	AGTCGCACTAGCCACATT	18	27	GAGAATCAAAGCCACCATC	19	532	506
pepQStrrmut	2323340	*S. mutans*	TGATTAAAGGAGTTAAGTTGGTG	23	699	TGTCAAAATTAGCACAGATTG	21	1079	381
perMStrmut	2952523	*S. mutans*	TTTTATTGGGAGGTTTTCTTT	21	134	AACAAATATGAAGGCAGCA	19	696	563

gene	gi	source species	forward	length	position	reverse	length	position	product
pflCStrmut	2952523	*S. mutans*	AGAAAAAGTTGACTACGAAAAAG	23	6	AGATAAATAACGCGCACAAG	20	510	505
pflCStrpneu	15459462	*S. pneumoniae*	CTTGTGCTGTTCTTCGTTG	19	337	CTTGCGCTACCGTGGTTT	18	586	250
plpA	1658316	*S. pneumoniae*	TTGATATCCAACAACTACAAAAA	23	524	AGCTTTTTGGTATTCATCTACAG	23	1023	500
ply	47403	*S. pneumoniae*	ATTTCGAGTGTTGCTTATGG	20	709	GTAAAGTGAGCCGTCAAATC	20	1226	518
prtA1Strpneu	9943836	*S. pneumoniae*	AGGAAACAGAAAATAAAGAGAAAC	24	143	CAAACGGAGCATTGATAGAC	20	667	525
prtAStrpneu	13345020	*S. pneumoniae*	GGGTAACTATGCGACTTCTG	20	1134	AGCTTTACACCATCATCTCC	20	1667	534
pspA	153840	*S. pneumoniae*	GATCAACAAGCTGAAGAAGAC	21	1179	CATCTTTCACCCAACCTG	18	1782	604
pspC1Strpneu	6469846	*S. pneumoniae*	CATTGAAACCAGGAGAAAAGG	21	401	TTTTTACTCTCAACTTTCTCTTTTGC	26	650	250
pspC2	6469844	*S. pneumoniae*	AAAAGCGAAAGTTAAGAGTGAA	22	600	GGATGGGCTTGGAAGAGT	18	879	280
purRStrpneu	15459462	*S. pneumoniae*	AAGATATTGCCAACCTCAAC	20	17	GGTTATGACCGTAGCAACTAA	21	430	414
pyrDAStrpneum	5578890	*S. pneumoniae*	GCTTGTATGACGATAGAGGAG	21	70	TAGCTCAGTCAGACCACGA	19	381	312
recNStprmut	24376948	*S. mutans*	TGATGCTGAGTACAAGGATTT	21	780	CTTTAAAATCAGCCTTATCCA	21	1186	407
recNStraga	22533503	*S. agalactiae*	GCATGACCAAGAGGAACTAA	20	390	TGTAGTAAACCAGCGTCAAA	20	899	510
sloStrep	13699903	*S. dysgalactiae*	TGTTAATAGTAAAATCTTAGATGG	24	813	CCGCTTCAACATCATTAC	18	1099	287
SP0828Strpneu	14972300	*S. pneumoniae*	TTGTAGGGCTAGGAACAGG	19	62	AATCTGAACTCCATCTCGTC	20	660	599
SP0830Strpneu	14972300	*S. pneumoniae*	ATGACGCGCTATGCTTTG	18	1	ATAGGCAGTCTTAGAATAGGATTCTTC	27	450	450
SP0833Strpneu	14972300	*S. pneumoniae*	AACCATGTATTGAGGAAAGTG	21	85	TGGAAGTTTGTATGAGTGACAG	22	611	527
SP0834Strpneu	14972300	*S. pneumoniae*	TGTTCGGATTGAGCTAGAAG	20	42	TAATTTTACGGTGGGGTTG	19	460	419
SP0834Strpneu	14972300	*S. pneumoniae*	GTGTCAGCACAAATTACGATT	21	1	TCGTCACTCACCTTGGAA	18	500	500
SP0837_38Strpneu	14972300	*S. pneumoniae*	GGACTTCCTTTTCTTATCCAG	21	13	CAGTCCATAGAGGCTGTTTT	20	573	561
SP0839Strpneu	14972300	*S. pneumoniae*	TTTGCAGATTTGTGAGATTG	20	90	TACGTTTACAGATGCTACGG	20	592	503
sphtraStrpneu	2109442	*S. pneumoniae*	ATCTGTAGAAGGTCTTGGTTTC	22	789	ATTCTAAATCACCTGAACTCTTG	23	1189	401
SPy0382Strpyog	13621628	*S. pyogenes*	CACCAGACAACCTTTCTTTC	20	30	CAAGTGTAACCAGAGCTTCC	20	571	542
SPy0390Strpyog	13621628	*S. pyogenes*	TTAATTGCAGTAGCAGCATC	20	916	AGCCACCAATAATGAGGAG	19	1531	616
SpyM3_1351	28876381	*S. pyogenes*	AGCAGTAATCTTTGGTACTGTTT	23	170	TGGTGAAGATAGATGAGATACTTAAT	26	377	208
ugdStrpneu	33113982	*S. pneumoniae*	AATAGCAGTAGCAGGGACAG	20	6	TGCTCCGTATCTCTCAAATC	20	506	501
uncC	18653430	*S. pneumoniae*	ATGAATTTAACATTTTTAGGCTTAT	25	1	TATTTGATAATAAATGAGAAGACAAGA	27	200	200
vicXStrepneu	6689277	*S. pneumoniae*	ATGAGTGAAATAGGCTTTAAATACA	25	1	CACCTTGCCAAGATATTTACTATT	24	300	300
vicXStrpyog	6689351	*S. pyogenes*	TTATCTGATTTAGGACATTTATC	23	580	ATATCAGTTAATGGACAAGCAG	22	809	230
wchA6bStrpneum	13377419	*S. pneumoniae*	TTGCACTTTATATCCTCCATT	21	116	GTTACATCAATTCCCATCGT	20	665	550
wci4Strpneum	13377403	*S. pneumoniae*	AAGTAGGGGCTTTCTTGC	18	290	AACTTTCTTTCTTACCACTACCC	23	634	345
wciJStrpneu	33113982	*S. pneumoniae*	ATTTGTGAGGATCTTGTTGAG	21	58	GGTATTCTTCTCTATCAAACTGG	23	747	690
wciK4Strpneum	13377403	*S. pneumoniae*	TAACGAGATTATTACAAAACAAAAC	25	120	TCTTTAGGCTGGAATGTGTC	20	719	600
wciL4Strpneum	13377403	*S. pneumoniae*	GAAAGAATTGGGTGCAAAG	19	150	TCATCCTCACCATTACCAAC	20	710	561
wciN6bStrpneum	13377419	*S. pneumoniae*	GCTTCCATCAAATCACTTTAC	21	49	GTGCAACATTACTGCCTTC	19	784	736
wciO6bStrpneum	13377419	*S. pneumoniae*	TGGAAGACCTTTATCCTGTC	20	96	AAAATAGTTAATAGCAGTAAAACCTG	26	620	525
wciP6bStrpneum	13377419	*S. pneumoniae*	CTATAATGGTGAGCGATATTTG	22	33	AGAACAACACCTTCCCATAC	20	628	596
wciY18Strpneum	13377447	*S. pneumoniae*	CGTATCAAGTCGGCATTT	18	528	GCAATTTATCAAAGTTATCATCC	23	1057	530
wzdbStrpneum	13377419	*S. pneumoniae*	GGATATGCCAGCAAAAAC	18	306	TGTCAAAATCTGGAACGAG	19	685	380

gene	gi	source species	forward	length	position	reverse	length	position	product
wze6bStrpneum	13377419	*S. pneumoniae*	ATCAACGACTTCCACCAA	18	147	AATATTTACGCAAGGTTTCAAG	22	436	290
wziyStrpneu	33113982	*S. pneumoniae*	CAACTTCTGATTATGCCTTTG	21	130	TCCTAATGTCCGTCCAATC	19	717	588
wzxStrpneu	33113982	*S. pneumoniae*	AGTGGAACTATGTGTTTAATCTTG	24	29	GATGTCAGCAACCATCCTAC	20	767	739
wzy18Strpneum	13377447	*S. pneumoniae*	AGATTACACTTTTACAGCTATCTCC	25	279	TCAACTCCCTTCCATAAATC	20	785	507
wzy4Strpneum	13377403	*S. pneumoniae*	TCTGGACTCTCGATAATTGG	20	71	AGGAGAACACACCAGGAAG	19	628	558
wzy6bStrpneum	13377419	*S. pneumoniae*	ACATTTGTTATAGTTTCCTTGTTG	24	202	TTTGGTAAGTTGAGATTTAGGAC	23	717	516
xpt	4495149	*S. pneumoniae*	TTTTAACCCACCAAGTTGAC	20	2	GATACCGATAGCTTGGACTG	20	384	383
ytqBStrmut	2952523	*S. mutans*	CGGCAGACAAGTCAGTCA	18	2	TATAATTTCTCTATCATCACCAAAA	25	251	250
ABC-eltA	15418957	*E. faecalis*	CATGTATTGGTTGTTAGATAGGG	23	204	GAGGATAAGGCCATGACTG	19	740	537
ace	10863251	*E. faecalis*	TAGTTGGAATGACCGAGAAC	20	128	AGTGTAACGGACGATAAAGG	20	717	590
agrBfs	6492218	*E. faecalis*	GATCAGGAAGATCAATCAGG	20	40	CTTAGTTCCCACACCATCAC	20	645	606
agrCfs	6492218	*E. faecalis*	GTTGGGCTACTCTTGGTTG	19	153	GTGTTGATACGTTGTTCTTCC	21	659	507
arcA	14349113	*E. faecalis*	ATATCGAAGTGGTCTATTTAGAGG	24	188	TTGATTCTGAACGATCATAGACT	23	637	450
arcC	14349113	*E. faecalis*	AATTAAACAAAGCAGGAATCA	21	300	CCACCACATGAAATGGTAA	19	579	280
asa1	47047	*E. faecalis*	CAGGAGGAACATTGGTTGT	19	1007	CGTCATACCCTGGATTGTT	19	1591	585
asp1	43323	*E. faecalis*	CGACAGAACTTGCTAAAGTAGA	22	1859	GTGATTCCGCCATAGTTG	18	2402	544
bglB	4704705	*E. faecium*	TCTTCATTTGTTGAATATGCTG	22	213	TGGAATCGAACCTGTTTATC	20	712	500
bglR	4704705	*E. faecium*	ATTATTGTCGCCTCTTTCC	19	7	GCTATTTGTTATCGCATTCTG	21	671	665
bglS	4704705	*E. faecium*	CTCCTGATCCTCTTCTTGTG	20	502	ATGTTATACTCTGCTCTTCAACC	23	1005	504
bkdA	5901691	*E. faecalis*	AGTTGCACAAGTAGCGATG	19	156	TGAGAAGTCAAGCGAGAAAC	20	755	600
cad	19481589	*E. faecalis*	CGCAGACAAGAAAGACAAC	19	72	GTGTGGTAGCCGTTTGAG	18	599	528
camE1	20522053	*E. faecalis*	TTTTCACTTTTAGGAGCTATTTTT	24	28	CTTTTTCATCTGCCGTAGTC	20	478	451
cgh	30058650	*E. faecalis*	CTTCTTTCGTGCTTTCAAC	19	507	AAATTTAGATCATCATTTTGC	21	847	341
csrA	5457307	*E. faecalis*	CAAAACCAACAGAAGAAGAATTA	23	5	CGAACTTCTACTCGGTGCAT	20	278	274
cylA	30058650	*E. faecalis*	AAACCTGGATGATAGTGATAGG	22	201	GACTCATTTCCTGCTGATG	19	794	594
cylB	30058650	*E. faecalis*	CAAGGAGAGCATAGTGAATGT	21	25	TGCAATAGGAATTAAGAGTAATAAAA	26	528	504
cylI	30058650	*E. faecalis*	GTTAGGGCACTTAGCTTTTG	20	120	TGGGTATATTAGTTTTAATTTCCTATT	27	681	562
cylL_cylS	30058650	*E. faecalis*	TGGAAAATTTAAGTGTAGTTCCT	23	2	CTACTCCTAAGCCTATGGTAAAAC	24	403	402
cylM	30058650	*E. faecalis*	AGCAAAGTGGTAACGAGAAG	20	56	ATACGCATCAACTCAGGATAG	21	617	562
dacA	12188895	*E. faecalis*	GGCTTAGTTGTCAGTTGTGG	20	61	AATTCCTTGCATTTGATACAG	21	624	564
dfr	4103866	*E. faecalis*	GTTTGATTGTTGCGAGGT	18	8	AATCTACTCAATTCATTTTTCCTT	24	485	478
dhoD1a	30025891	*E. faecalis*	TTTTACTAAACCATTAGGTGTAAAAT	26	501	CAAAAACTTGTGGACCTTCT	20	880	380
dnaE	21314373	*E. faecalis*	TTACGTTAGAAGAAGCATACCA	22	1349	AGGACGATACAGGGCATT	18	1848	500
ebsA	388106	*E. faecalis*	TTTATGAAGGCCCAAAGA	18	31	AGCCAGAATTAGCATCAACA	20	437	407
ebsB	388106	*E. faecalis*	TTTACCTCACCGACCAATC	19	65	TGGCTGAAGAAGCTCAAC	18	671	607
eep	5714509	*E. faecalis*	TCTGTTTACGTTAGCGGTCT	20	564	TCCCTAAGTTCATTGACAACA	21	1084	521
ef00108	21693259	*E. faecalis*	TCAACGTCACAAACAAGAAC	20	33	CCTAAGAGACTTCCTTCATCC	21	680	648
ef00109	21693259	*E. faecalis*	TCGGAAGTATTGCGTTTG	18	98	TTATCGACATTCTCTGTTTCC	21	617	520
ef0011	21693259	*E. faecalis*	TTGTCTTTGGGCTTCTCTC	19	17	TTTCTGTATTGCTTCTTGGTC	21	684	668

gene	gi	source species	forward	length	position	reverse	length	position	product
ef00113	21693259	*E. faecalis*	CTATAGATTCCCTATCTTGTTGG	23	69	ATATTTACCGCAATGAAACAC	21	585	517
ef0012	21693259	*E. faecalis*	ATGTATGTATCTTTTGTTCTAAGTTGT	27	1	AAATCCCAATCGTCTAAGTG	20	380	380
ef0022	21693259	*E. faecalis*	AAAGTACTACCTTTTATTGCCTTAGT	26	7	TGTTTTATAAGTGTTCTTTGTTTTTC	26	326	320
ef0031	21693259	*E. faecalis*	TGAACAAAAAGCACAGGATAG	21	78	TACTAAACCCCCGTTGTG	18	580	503
ef0032	21693259	*E. faecalis*	TGATGAAAATTTAAAAGAAGAAGC	24	39	TAAATCCTCCATTAAATCCTCCA	23	258	220
ef0040	21693259	*E. faecalis*	TCAAATACACGTAAGCCTTCT	21	491	AAGTTTTCATTTCGAGAAGTTG	22	870	380
ef0058	21693259	*E. faecalis*	CATTATTTTCATTAGGGGATATTAG	25	146	AGATTCCTTACTTTAGCTTTTCC	23	656	511
efaR	15425977	*E. faecalis*	GGCGACGAAGTTAAAGTG	18	49	AATGGTGATCGGTCCTTC	18	579	531
efmA	3790617	*E. faecium*	CGGATGAAGGAAGTAAAGAA	20	83	CATATGCTCGTAAATACTTGAAA	23	637	555
efmB	3790617	*E. faecium*	AAAGCGATTTGTTGCTGA	18	398	GAAAATTAAATTAGCAAGGATAAAA	25	798	401
efmC	3790617	*E. faecium*	AGCCGGTAAACTCAGTCC	18	197	CACCATAAGGCAATTGAAG	19	703	507
enlA	11078548	*E. faecalis*	TTGCGATTTCTGTTGTAGG	19	18	AACCAAAGTCAAACCCATC	19	543	526
entA_entI	7271910	*E. faecium*	TCTATTAAACAGACACAACTTATCTAT	27	22	CCGGGATCTAATTCATATTT	20	342	321
entD	4760901	*E. faecium*	TTCATAAGGACGATGTGTTG	20	242	TGTTCTAGCTCTTGCTTTCC	20	854	613
entR	4760901	*E. faecium*	ATTATGTGAAGATCAAATTATACA	24	18	TTTTGTTGCATCTATCCTTTC	21	417	400
esa	493016	*E. faecalis*	AATCAAGCCGCTGAAAAG	18	64	CAAATTTAGCTTTGGCTTCC	20	568	505
esp	30058650	*E. faecalis*	AAATGGGTGAAGGAAGATTAG	21	149	ACCAGTGCTTTCTATTCCAG	20	705	557
gelE	1339878	*E. faecalis*	AGGTACAGGCATCTTTGTTG	20	30	TTCCATAATTGTTCCATCTGT	21	552	523
gls24_glsB	2274939	*E. faecalis*	GATACGAAGAAGATAGCGAAA	21	404	ACAATAGCCATCCCAGCTA	19	745	342
gph	9187965	*E. faecalis*	AAGATGGTACGTGTATTCGTTT	22	29	AATTACTTCTGTCGCCTTCA	20	540	512
groEL	15625348	*E. faecalis*	CTGGTTCAAAAGAAGCCATT	20	1001	TTTAGTTTGTCAACAATCACTG	22	1400	400
groES	15625348	*E. faecalis*	GATCGCGTCGTAATTAGAGT	20	19	TTCAACAGTGGCAATAATGT	20	282	264
gyrAEf	15982566	*E. faecalis*	TTATTTCTGTTGAGGGCAAA	20	1802	TCTTCTTCCACTTCTTCTGG	20	2444	643
H+ATPase	15667455	*E. faecium*	TTGGCAATGCGATGTTAG	18	29	CGCGATTTGAAGAGAGAG	18	429	401
metEf	2353762	*E. faecalis*	TTGATCGTTTTGACGTAATG	20	26	TAATCGTTTCTTTCTCTTTGG	21	427	402
mntHCb2	29134879	*E. faecalis*	AGCGACTAGAGAGCATACAAG	21	309	TCAGAGCATCAAATAAATCAAC	22	916	608
mob2	4127803	*E. faecalis*	AAATTGTTGCACGTATGGA	19	8	TTTGCCGACTAAACACCT	18	457	450
mreC	9246947	*E. faecium*	ATTACTCGTTTCCCCTGAC	19	6	ACAATCGTCACAACTGACAC	20	461	456
mreD	9367034	*E. faecium*	ATGTTGAAGAAAGAAACAATGA	22	1	CTGAATAATTTCTTAAAGGGAAAA	24	500	500
mvaD	9937385	*E. faecalis*	CAATGGAACAAAGGCCACT	19	451	TGCTTGGCGAACTAAAGC	18	810	360
mvaDEfaecium	9937392	*E. faecium*	TCAGTCAGTTTCTTGACCTTT	21	215	TTAGTCCGTTTCTTTCCATC	20	721	507
mvaE	9937382	*E. faecalis*	CTGCGGTTAAAGTCGTTG	18	261	AATCACGTAGCCTTACATCC	20	786	526
mvaEEfaecium	9937389	*E. faecium*	CCAATTAGGTGAAGCAGAAC	20	306	TTTGATGGTTGCTAAATAAGG	21	813	508
mvaK1Efaecium	9937392	*E. faecium*	GATTGCCTTTCCTTTCTATG	20	81	ACATCTTTGACGGCTTCTC	19	581	501
mvaK2Efaecium	9937392	*E. faecium*	CAAGAACAAGAAACTCAGCA	20	550	CATGTAACGGCAGTGGAG	18	1054	505
mvaSEfaecium	9937389	*E. faecium*	AAATTCACTTACTGCACCAGA	21	214	ATGGCGAAAGAATATGTCAA	20	798	585
oep	9246947	*E. faecium*	ATCTTGATCTTGCCATTCC	19	775	TGCTTTCTCAATCTTCGTTT	20	1307	533
orf3_4Efaeciumb	4704705	*E. faecium*	GCATATTTCGCTTGATATATAGGT	24	243	TTATTCATGCTGGATTATTCG	21	542	300
orf6_7Efaecium	3342117	*E. faecium*	ATATTTCATCCCCAGCTCTT	20	19	AAGTATGAAAGAGATAAAACAAACAT	26	291	273

gene	gi	source species	forward	length	position	reverse	length	position	product
orf7_8Efaecium	4704705	*E. faecium*	AGCAGTTCCGGTATCTCTTT	20	53	TGAAGAAAGGGTAATGTTTTG	21	678	626
orf9_10Efaecium	4704705	*E. faecium*	TGAAGTCCTTTGTCTTTGTTG	21	7	CAATACACACCGATTTAGAGG	21	514	508
parC	15982569	*E. faecalis*	ATCCGACTATGCGTTTACTG	20	1236	TATGTTCGCCAATTTCTTTC	20	1747	512
pcfG	30841312	*E. faecalis*	AGGATCAATCGTAAATGGTG	20	87	TTCGCTGCAATCTTATCC	18	690	604
phoZ	5020413	*E. faecalis*	AAGATGGTGAAACATTGGTG	20	947	TCTTTAATCTCAGCTTTTTGC	21	1397	451
polC	21314371	*E. faecalis*	GTTTCCGTTCAAATAACCAC	20	193	CAATTCACGCACTTCTTTATC	21	735	543
ptb	5901691	*E. faecalis*	AATTGCAGGAGGTTCACA	18	15	CTTCAGAGGTCGCTAAATCA	20	592	578
recS1	30058650	*E. faecalis*	AATGCGTGATCAGGGTGTAT	20	20	TGTAATTATGAGTCGAACATTTTTATT	27	339	320
rpoN	12003288	*E. faecalis*	CTCAACAGCTTCAACAATCC	20	59	CCACTATTCGATCTTCCTTG	20	751	693
rt1	27262899	*E. faecalis*	ATCTCGCGGAACAATTAGA	19	41	AATAAGCCAATCCACATATCA	21	545	505
sagA	9367034	*E. faecium*	TGAATCTTCAGCAACAGA	18	1017	TTTGGTTGTTCAGGTGTT	18	1187	171
sala	14906073	*E. faecalis*	TCGGCTCTAATGGTATGTTC	20	19	GTTGACGTGCTTGTTCTTC	19	706	688
salb	14906081	*E. faecalis*	GCTTCATTAGCATTAGAACAATC	23	601	AAACCTGCATCCCATACTAC	20	1100	500
sea1	49019	*E. faecalis*	TTAACTGAACAAGAAAAGCAAG	22	964	TTAAATCTCCTGAAATAGAAACTG	24	1492	529
sep1	1272652	*E. faecalis*	ACAACCAACAGTGAAAGCTAC	21	126	TTGTTAGCCAATTCTGCTTC	20	683	558
tms	4103864	*E. faecalis*	AGGTTTAGAAGTGGGGGAGT	20	245	ATCGGCGCTTTAATCGTT	18	454	210
tyrDC	21309881	*E. faecalis*	AACGCACATCTGAAAGCTAC	20	173	TACCTGAACGAGCTGAATG	19	676	504
tyrS	21309881	*E. faecalis*	ATGGAGGGTGATAACATGAA	20	1	AAGATAGTTCTGATAACCAATCG	23	400	400
vicK	6687468	*E. faecalis*	CATTGGTGGCTATTTCATTC	20	93	CTTTCTGCTTCCATTGTTTC	20	779	687
yycH	6687468	*E. faecalis*	TATCTTAGCTTCGCAACCAG	20	231	ATTGTCTGCCACGTATAACC	20	764	534
yycI	6687468	*E. faecalis*	GAGCAACGTCTCTTCTTCC	19	156	GAGCGTGTCTATCGCATC	18	702	547
yycJ	6687468	*E. faecalis*	TTCATCGCAATAATCGTTC	19	410	TAACACATCCGCCATCAC	18	633	224
b1169	16127994	*E. coli*	GTTTGGGACTTATTGCTCTG	20	29	CATCAGCCACAGTTTCAAG	19	775	747
b1202	16127994	*E. coli*	GAATACCAAAGCAGATCGTC	20	24	CCGAGATCGACAACAGAG	18	758	735
eae	145852	*E. coli*	CTAACTCATTGTGGTGGAGC	20	28	CTTGTCATCGGTCATGTTG	19	536	509
eltB	145830	*E. coli*	GGCGTTACTATCCTCTCTATG	21	33	TTTCCATACTGATTGCCG	18	370	338
envZ	453286	*E. coli*	AGCCTGGTGACGACTTATC	19	76	ATCCGCCAGTTGCTTAAC	18	696	621
escR	2897961	*E. coli*	TTTGTTGTTATTGGTACTTCATTC	24	79	ATCGAAATTGTTACTGGCG	19	578	500
escT	2897961	*E. coli*	TTACGCTTCCGATCATAGTAG	21	137	GAATACGTTTAGTTGAGGCG	20	639	503
escU	2897961	*E. coli*	AAGTGAAGAGGTAATGGCTG	20	72	TACCATCAGTATCCTTGGC	19	688	617
espB	1657262	*E. coli*	GATGGTGACTCTATTGCAGG	20	174	CCATACGATTCTGGACCTC	19	913	740
fes	145916	*E. coli*	TGTTTGAGGTCACTTTCTGG	20	2	CAATAGCTTTCACCAGGG	18	718	717
fes	145916	*E. coli*	TGTTTCTGCACTCGAAATG	19	342	GGCAATAGCTTTCACCAG	18	720	379
fliCb	8071787	*E. coli*	ACCACGACAGGTCTTTATG	19	598	AGAGAGGCACCGTCACTAC	19	1046	449
fteA	1621225	*E. coli*	TATGCTGCTCCAACTATTCC	20	25	ATTATCTCCATCTTTCAGGG	20	426	402
hlyA	525328	*E. coli*	CTTGGAAATGTTGGTAAAGC	20	77	TAAACTCCTTCGGTTGAGC	19	800	724
hlyB	1247757	*E. coli*	TCAATGCTGAAACTATAAGGC	21	74	ACTTAGCACCCAGTTCGAC	19	691	618
iucA	474189	*E. coli*	CATCAGGCAGTTATCCTGTC	20	215	AGTCGTCCTCCTGCATTAC	19	982	768
iucB	474189	*E. coli*	TTCACAGCGGATATGGAC	18	20	CACTTTGCTCCCAGAAATAC	20	520	501

gene	gi	source species	forward	length	position	reverse	length	position	product
iucC	474189	*E. coli*	AGACTGGGATTTGGTCAAC	19	12	AGACACCATCCTGCCTTC	18	732	721
nfrB	16127994	*E. coli*	ATGGAATTGCGTCTGTTC	18	529	AAGTTTAGCCACAGCAGG	18	1232	704
nlpA	146648	*E. coli*	ATAATCCTCGTCATTTGCAG	20	518	GACTTCGGGTGATTGATAAG	20	768	251
papG	42307	*E. coli*	GGAGTATATTGCGTGGGTAG	20	234	AAGATTCACCATAGAGGCG	19	952	719
pilAe	147269	*E. coli*	CTCTGTCCCTCAGTTCTACG	20	38	TGGTATTGGTTCCGTTATTC	20	460	423
rfbE	1911762	*E. coli*	AAATGAATGTCTGGACTCAAC	21	57	TGTAAACAACGGTCATAAAGTG	22	617	561
shuA	21954703	*E. coli*	AGCAGCATCAGGTTCTTG	18	23	CAGTTGTTCAAACGCGAG	18	779	750
SLTII	304950	*E. coli*	TTCTTCGGTATCCTATTCCC	20	48	TGTGAGGTCCACTTCTTCC	19	663	616
toxA-LTPA	148027	*E. coli*	AAATGGCGACAAATTATACC	20	54	CTGGGTCTCCTCATTACAAG	20	666	613
VT2vaB	148261	*E. coli*	AAGAAGATGTTTATGGCGG	19	4	GATTCACAGGTACTGGATTTG	21	230	227
yacH	16127994	*E. coli*	GACTCGGTACAGCGATTG	18	517	CTGACGTTGGGTATCTCG	18	1284	768
yagX	16127994	*E. coli*	CTTTACGACGGTTCTCCC	18	5	AATCTTCCCTGCTGAAATG	19	792	788
ycdS	16127994	*E. coli*	TTGAAACTTCTTACTGCCG	19	46	AATTTCTAATGCAGCGTATTG	21	795	750
yciQ	16127994	*E. coli*	ATAGCAGGGCTGTTTGTATC	20	34	GACACGGAAACCAAATTAAC	20	766	733
ymcA	16127994	*E. coli*	TATTGTCATCGCGCAGAG	18	541	TGTTGGGTTGAAAGAGTAGC	20	1296	756
acoA	501025	*K. pneumoniae*	TAACGGCAAAGACGCTAA	18	380	TGACCAGGGCTTCTACTTC	19	880	501
acoB	501025	*K. pneumoniae*	CCTATAATATCTTTGCCACCA	21	440	CCAGGGATCCAGAGTTTT	18	974	535
acoC	501025	*K. pneumoniae*	GCTTGAAATGCCAAAGTG	18	18	CAGATCAGAAACAGAGATACGA	22	560	543
ahlK	31540968	*K. pneumoniae*	CAGTCAGGAACACAGCATT	19	31	TCTAAGGTATAAGCCGCATC	20	626	596
aldA	29243337	*K. pneumoniae*	GGGGAGAATATCCTTGTCTT	20	397	GCAGCGGCCATAATTTTT	18	722	326
aldA	29243337	*K. pneumoniae*	GACTCGCGGGTGATTAAC	18	823	CAGTCCCTTAATCGCTTTC	19	1287	465
atsA	7963895	*K. pneumoniae*	AGGATCATTTGTCTCCTACG	20	1080	ATGGTGCGCTTAGCAAAT	18	1598	519
atsB	7963895	*K. pneumoniae*	CTGATCAACGACGCATGG	18	361	GCGCTGAGCTGGTATCCT	18	680	320
budC	3907615	*K. pneumoniae*	CCGATCGAGTCCATTACC	18	280	GCGTACGGCGAATTTACT	18	480	201
cim	6176537	*K. pneumoniae*	AATTTAACCTGGTTTGATAAGAA	23	16	CAAAATATGAACTATCAGAAAGATTG	26	226	211
citA	924990	*K. pneumoniae*	GCCTGCTTCGTTGATAGATT	20	1261	ATACTGCCGCCTGCTTGT	18	1565	305
citW	18140905	*K. pneumoniae*	GCCACCTTTATTCCTTCC	18	289	AGCATCCCCATCATATACAG	20	896	608
citX	18140905	*K. pneumoniae*	GTCAGCATCGAGGCATTG	18	31	CGTCAATCATCTGCTCAAC	19	520	490
cymA	854227	*K. oxytoca*	CTCTGCCTCTATTGCTCTTG	20	18	GCACTATATCCCTGCTCTTG	20	602	585
cymD	854227	*K. oxytoca*	TATCGATGCGGATGAAAAT	19	512	TTAATATAACCTTCAAGCAACACT	24	851	340
cymE	854227	*K. oxytoca*	ATCCAATGACCAGAAATGAG	20	210	AGATATGCGTAATCCACAGG	20	817	608
cymH	854227	*K. oxytoca*	CGTAAATATGGGACAAAGGT	20	1132	TAGATTTCGCACAGTCAAAG	20	1720	589
cymI	854227	*K. oxytoca*	GCAAGGGTAGAGGTGTATTG	20	8	TGGCTGTGAACTTTGTGTAG	20	535	528
cymJ	854227	*K. oxytoca*	ATAAGCCATGTGTTTCTTCC	20	174	CACGTTCTAGCGTCTACTCA	20	683	510
dalD	2905644	*K. pneumoniae*	GTTGTTCTCCACTACCCACT	20	637	AGATCACCTCAATCCAGAAA	20	1145	509
dalK	2905644	*K. pneumoniae*	GACTTCCGGTTTTTCACAC	19	106	CGGTACATGCGTTTTGTC	18	638	533
dalT	2905644	*K. pneumoniae*	CCAGCTCGGAAAACTTCT	18	636	GGCGATTGTCCATAACGTC	19	895	260
ddrA	3115375	*K. oxytoca*	GCCGGTAATCTTGAGCTG	18	781	CCGATCTGCACGTCGATA	18	1163	383
fdt-1	1304134	*K. oxytoca*	CCTGCTCTATTCCGTCAG	18	5	GATTTCCTGTACCCGGATG	19	404	400

gene	gi	source species	forward	length	position	reverse	length	position	product
fdt-2	1304134	*K. oxytoca*	GGCTGTCTGCTATGGATTT	19	85	GGTCGATCATCAGATTGG	18	619	535
fdt-3	1304134	*K. oxytoca*	TGGCGTTTATTTTCTGGA	18	2	GTAGCGTTGCATGGGATA	18	401	400
fimK	349480	*K. pneumoniae*	CCGTTACCGATGTTGATT	18	52	GAGTGAGGAGGGTATGTTGA	20	744	693
gatY	18139839	*K. oxytoca*	CGTTCTCCCCTGATTCTT	18	124	TCATAGCGGTGGCTCAGT	18	383	260
glfKPN2	765057	*K. pneumoniae*	CCGATCATGAGAACATCAG	19	617	GGCATCTCTTCCTGGTTAG	19	1130	514
hemly	24305989	*K. pneumoniae*	ATGAAACGACCTGATTGC	18	1	GTGTGGACCGAAGAATTG	18	465	465
hydH	12584123	*K. oxytoca*	CGGCGCATTTAAAATATC	18	908	CATTAACCGGCAAATAGAAC	20	1363	456
liac	19568920	*K. pneumoniae*	CCTGCTGCTATTGCTGTC	18	39	GTATGGTGCCAGATGCTC	18	728	690
ltrA	924990	*K. pneumoniae*	AGAGATGGGCTGCAAACT	18	132	CGATCCCTAAATGCTGTCT	19	715	584
masA	392019	*K. oxytoca*	TATTGAAGGCACCACCAG	18	24	GATGAATATCGGACAGGAAC	20	559	536
mdcC	2240010	*K. pneumoniae*	ACAGATTACATTGTCATTTCCTG	23	6	CCTCCTCAAAAACCTGTTC	19	286	281
mdcF	2240010	*K. pneumoniae*	ATGGATTTTGCCTTACCC	18	121	CCAAGTAACATCACCAGACC	20	776	656
mdcH	2240010	*K. pneumoniae*	TATTTACCTTTCCCGGTCA	19	11	CAATCACGAACTGCGTTT	18	466	456
mrkA	149187	*K. pneumoniae*	TGCTGCTGATACCAATGTAG	20	63	GTAATTTCGTAAGTCGCGTAG	21	599	537
mtrK	21264676	*K. pneumoniae*	CGCAATACCATACCTTCAC	19	5	CTCGTGGATCTGATACGG	18	558	554
nasA	4755081	*K. oxytoca*	CCTGGAGTGTGCATAAGG	18	1610	CAGCGAAAGATGGATAATG	19	2166	557
nasE	2642135	*K. oxytoca*	ACACGCATATAAACCGCAAC	20	6	GAATCCAGCGTGGAGAGC	18	236	231
nasF	2642135	*K. oxytoca*	GCGATAAGTTTTCGATTTCA	20	5	GCTGACCGATCCGTTTTT	18	463	459
nifF	43820	*K. pneumoniae*	GTGCGTTTAATCTCCTCAAG	20	11	GCTATCCGGACAACTTCG	18	230	220
nifK	43820	*K. pneumoniae*	AAATTGCCGAAGCTCAAT	18	649	CTGACGGTCATCAGCAGT	18	989	341
nifN	43820	*K. pneumoniae*	AGAGAGCGTCATTGAGCA	18	450	AATTTTCGCCAGTTGATG	18	849	400
pehX	17978816	*K. oxytoca*	ATGTCATGGTTCCGATACTG	20	134	CTTTATCAAGCGGATACTGG	20	727	594
pelX	17981479	*K. oxytoca*	AAGCTGGAACGTACTAACGA	20	670	TAATCACTGCGGCTAATACA	20	1294	625
pSL017	7328180	*K. pneumoniae*	TTGCGTATATAGAAGTCATACCA	23	10	GCGTGAGCTGTCTACCAG	18	279	270
pSL020	7328182	*K. pneumoniae*	GATTCCTGCTCTGACAACC	19	6	GCCTTGAATACTGGAAGAAAG	21	411	406
rcsA	24459262	*K. pneumoniae*	ATTGACGGGATATCTGACC	19	45	ACATTTGCAGCATATTTGATT	21	445	401
rmlC	22759339	*K. pneumoniae*	CAGCAGCAAGGTGTTTAATG	20	90	GCAGTGGCCAGAGTATGT	18	539	450
rmlD	22759339	*K. pneumoniae*	GGGGAGAAAGAGACCCTCA	19	511	TATTTGCCCAGGACTTCTGTT	21	890	380
tagH	18139839	*K. oxytoca*	ACAGGATAGCGAACACCTC	19	300	AAAGCCAGCTCCGTTTAG	18	802	503
tagK	18139839	*K. oxytoca*	ATCCATACCCTGACACTCAA	20	4	GATCAACAAAGGCCGTAA	18	537	534
tagT	18139839	*K. oxytoca*	GCTTCAGGTGTTGAAAATG	19	124	AGCAGCAGCATAAAATAACC	20	722	599
tyrP	6942303	*K. pneumoniae*	ACAGGGTTGATCCTCGTC	18	115	GTTAATGAAACGGCGAAAC	19	650	536
ureA	149330	*K. pneumoniae*	AAAAGACAAGCTGTTGCTG	19	21	CAGATAATCGGGTTGTGAA	19	302	282
waaG	5006979	*K. pneumoniae*	AGTAAATTCAGGCTGGCTCT	20	4	TTTACGTGGGTTACAGATATGG	22	183	180
wbbD	6137211	*K. pneumoniae*	TCATTTGAAGAACGACACAG	20	22	GTCAGGCCATATTGTTGG	18	710	689
wbbM	765057	*K. pneumoniae*	TGCCTCTATTATCCAACCTC	20	54	GACGATCCAGAATGCTAAAC	20	634	581
wbbN	765057	*K. pneumoniae*	GGTTTAAGGCAGGTAGTCAG	20	203	CCACGATACATGAAACACAG	20	851	649
wbbO	765057	*K. pneumoniae*	ATTTCATAAACTCGGATTGG	20	17	GGCAAACAGCACTACAGG	18	603	587
wbdA	6137211	*K. pneumoniae*	GTGGCATTGGTCGTTATAGT	20	50	CAAGGACGGACTCTTTCTC	19	655	606

gene	gi	source species	forward	length	position	reverse	length	position	product
wbdC	6137211	*K. pneumoniae*	TGACCTATCACTCGGATATTG	21	47	AGAGCTGGAAGAGAATGTATTT	22	496	450
wza	4808912	*K. pneumoniae*	AGGTTCAGGTAGCTGGAAAA	20	235	ATGATTCTGGGTTAGATCTCC	21	554	320
wzb	4808912	*K. pneumoniae*	TGATTCAATTTTAGTGATCTGC	22	6	AGGTGCTATCCGGCTAAT	18	285	280
wzmKPN2	765057	*K. pneumoniae*	ACATGATCCCGGAGAAAT	18	581	TTACAAAATCTCTGCAAATCG	21	780	200
wztKpn	3142231	*K. pneumoniae*	TTCAGGCGAAATGCTATG	18	536	TGGTTCCGTAGAGGAATAAG	20	1042	507
wztKPN2	765057	*K. pneumoniae*	TGGAACCAGTGATCAATTTC	20	2	GCTGCTGAACTTCCTTCC	18	388	387
yibD	5006979	*K. pneumoniae*	GCTATGAACTGATCCTGGTG	20	101	CGATATGGCGTACTTTATCC	20	778	678
yojH	2240010	*K. pneumoniae*	GGTGTCATCCCAAACCTG	18	124	GGTACCGAAGTGGTGGTC	18	348	225
aad	1015425	*P. mirabilis*	CGCTATTAACCTTGCTGAAC	20	207	CCTTTCTCACTCACCACATC	20	734	528
atfA	1504104	*P. mirabilis*	TGAAAAAGTTATTATTATCTGCAA	24	2	ATCAGTGGAAATAAGAGAAGC	21	501	500
atfB	1504104	*P. mirabilis*	AGATGAAAAGCTTGCTAATGA	21	333	TTAATGAACGATTTCTTTTT	20	678	346
atfC	1504104	*P. mirabilis*	TGGCTGACTCTCCTGATG	18	119	TTGCAGATAGGTATTGATATGG	22	681	563
ccmPrmi1	3395515	*P. mirabilis*	ATGGATAATAAGCGAACACAG	21	1	GACATACTGCCACAACGAC	19	527	527
cyaPrmi	726356	*P. mirabilis*	TTATATTGAAACACTGAAGCAAC	23	9	CTCCTGCCCATTGTTCTA	18	439	431
end	2673847	*P. vulgaris*	GTTCATAGGCTTCACGTAGTTC	22	1	GTCAAATCCATTACTTAGCACA	22	260	260
envZPrvu	2623217	*P. vulgaris*	CATCTTATTGTGGGTCCAAG	20	106	AAGAGCGAATACCTGATGAC	20	688	583
flaA	6959881	*P. mirabilis*	ATCACTTCTATCCAAAACGAA	21	325	AAGTTGCTAAAGCGTCATCT	20	844	520
flaD	6959881	*P. mirabilis*	CAACAGTGATTTCCATTTGAG	21	18	ACGCCTTGAACCTTTAGAC	19	532	515
flfB	1857436	*P. mirabilis*	TTGTTGCAATACAGCCATC	19	7	CGATAAATTACAAAATACGTTTCA	24	415	409
flfD	1857436	*P. mirabilis*	AACTTTAGCCCCACTACTATCA	22	16	AATACCATTATCGGGGAAG	19	528	513
flfN	1857436	*P. mirabilis*	ACTCCGCCAGACCTTAGA	18	12	ATATTGAACGCTGTGCTTGT	20	415	404
flhD	2384645	*P. mirabilis*	GCGATTCTATGGCTGATG	18	116	TTATGCCCGTTTCTTTGTAG	20	351	236
fliA	6959881	*P. mirabilis*	ACACAATCCATTCATCAGTTAG	22	301	TTTCGGGAAGCAATTCTAT	19	574	274
floA	1857436	*P. mirabilis*	TCTAATCTTAATTCCTCTGTTCCT	24	62	TGATATAGACTTGATTAAAGGTGATT	26	694	633
frdC	45917	*P. vulgaris*	CGTAAGCCTTATGTTCGTG	19	13	TAAACAATTAGCGCCACTG	19	395	383
frdD	45917	*P. vulgaris*	AATCAGAATCAACTTCCTAAGC	22	4	AACACCAATAATTGCGATAAC	21	348	345
ftsK	1922904	*P. mirabilis*	TCTTTACTGAGCTTCCATCC	20	118	CATCACTATCTGGCTCTATTTG	22	745	628
gstB	1053074	*P. mirabilis*	TATTGAGCGCATTGATTTAC	20	78	CTATGCACATTAGGACGTTG	20	578	501
hemCPrmi	726356	*P. mirabilis*	TCATATCGCTGTATTACCTTGA	22	1	CTTATTAGCCGCCCTTAAC	19	301	301
hemDPrmi	726356	*P. mirabilis*	AGCGCAAACTCTTCAGATAC	20	86	TTTAATCACTCGTCCTAGCC	20	592	507
hev	2394311	*P. mirabilis*	AATATTGGTCAAGCTGCAA	19	4	ATGTTCTGTCCCTGTTTGG	19	405	402
hpmA	150888	*P. mirabilis*	GACTTAATTGCTCCTCGTATTG	22	583	CGCGTCTAAGGTAACATCTC	20	1239	657
hpmB	150888	*P. mirabilis*	ATTAAGCGCAAATGAAACAG	20	54	GATATTGAGATGCCAAGGTG	20	675	622
infBPrvu	3850829	*P. vulgaris*	GCGAAGTAGAAGAGAAAGCA	20	359	TTTCAGAGTGGCGGTTATT	19	892	534
katA	8347010	*P. mirabilis*	AAATAGTGGTGGTGTGTTCC	20	1152	TTAGCGTCTTTCCCTTCTAAC	21	1451	300
lad	7007411	*P. vulgaris*	AGCTGATGTTGTTGTTGTTG	20	162	TTGATTGCACCTTTCTCTG	19	743	582
lpp1	150895	*P. mirabilis*	ATGAAAGCAAAAATTGTACTAGG	23	1	TTACTTGGTTATCTAGACGCTGA	23	220	220
lpsPrmi	14624978	*P. mirabilis*	AAAAATGTAGTGTTTCAGATTTTAG	25	23	TCATAGCGATACTTTATTATGTTTT	25	472	450
menE	8347010	*P. mirabilis*	TGCACTTGTTTACGAGAGATT	21	32	GAAATGGCCTCTACTGTTTG	20	552	521

gene	gi	source species	forward	length	position	reverse	length	position	product
mfd	3360265	*P. mirabilis*	CAGACAATGCGTTTATTTTG	20	6	TACGTAATGCGGCGATTC	18	278	273
mrpA	6453631	*P. mirabilis*	TAAATTAGCTTTAGTTCTTGGTTTAG	26	12	GTTTTGAGCAGCACTTGG	18	411	400
mrpB	6453631	*P. mirabilis*	TGGCACCACTATTGCTATG	19	14	TTTTGGCGTATCGTTATTTT	20	513	500
mrpC	6453631	*P. mirabilis*	GATGGTAATGCTGATAATAACAAA	24	1504	ACTAACGCGGTGTAATGCT	19	2049	546
mrpD	6453631	*P. mirabilis*	CAATCTATGCCTCGCAAA	18	431	AGCTGCCGTTTCTGTTACT	19	750	320
mrpE	6453631	*P. mirabilis*	AACAGTGGTTTAGCCCAAG	19	11	TGGCAACAAAATCTCCAC	18	517	507
mrpF	6453631	*P. mirabilis*	TTACTAAATTCTACAGCAGTAATGG	25	34	ATTGGTATTCTGCTAATAAGGTG	23	484	451
mrpG	6453631	*P. mirabilis*	CCGGTTCATATTGGGTCT	18	24	TCAGTCATAATTCAAATAGAAGGT	24	549	526
mrpH	6453631	*P. mirabilis*	CCTTCTAACGCCACTTACAC	20	109	ATGCGATAACCAACACTTTC	20	613	505
mrpI	6453631	*P. mirabilis*	AACATATGAGGGTGTGGACT	20	158	GAAGCAACGTAAATTTCTTACAC	23	565	408
mrpJ	6453631	*P. mirabilis*	TTGATTTTGCGAATATAGATGT	22	14	CAATAATGAAAAGCATCAGGT	21	323	310
nrpA	4097157	*P. mirabilis*	AAACAATAGATACTTTGCCACTT	23	8	TTCGCTGTTAATATCCCACT	20	776	769
nrpB	4097157	*P. mirabilis*	CTTGCCGTAGTTGAAGGTC	19	76	TACATTGGCATTAGCATCAG	20	591	516
nrpG	4097157	*P. mirabilis*	TTAAGTAAAGCTAAATCCAGAATG	24	73	ATAAATGATGTGGCAGGTTG	20	745	673
nrpS	4097157	*P. mirabilis*	ATTATTCTCGCCAGTGTTTC	20	1049	CGATACGTTTAGCACCTTG	19	1730	682
nrpT	4097157	*P. mirabilis*	AATGTAAATAAAGACAATGCTAACC	25	46	ATCTTGCCATGCTTCAAC	18	654	609
nrpU	4097157	*P. mirabilis*	TAAAGCATTCGGTATTCCAC	20	138	AGCGCATTATTCCATTCTAC	20	746	609
pat	3395515	*P. mirabilis*	AATTGGCTACTCCTCGATTAC	21	5	TGTTGTGCTAAAGGGTGTG	19	506	502
patA	14624978	*P. mirabilis*	TGGAGTATCAGAGCTATTTTGTT	23	34	AGTCAGCCATAATTTATCTAGTTTT	25	543	510
pmfA	515900	*P. mirabilis*	TGCGGCTTTAGTATTTGG	18	27	GAAGTTGATAACGGCTTGG	19	537	511
pmfC	515900	*P. mirabilis*	TTTCTCACAAGCAGAGCAG	19	72	TCATCGCTTTGTAAATTGG	19	797	726
pmfE	515900	*P. mirabilis*	ATGCCGTATTAGATCACACC	20	137	TTTCCCACATTACCAAAGTC	20	704	568
ppaA	2624374	*P. mirabilis*	AACAGGCACATTAACAGAGG	20	1146	TTTATCTTCAGGGAGCAATC	20	1689	544
pqrA	506865	*P. vulgaris*	TGGCTGAAAATGTCGTTAAT	20	2	AATGATTTAGCCGCTTCT	18	200	199
putA	6959881	*P. mirabilis*	CTATCAGCCACCTCTTCTTG	20	1172	CTAATGAGCATCGTTTAGCC	20	2105	934
rsbA	3320463	*P. mirabilis*	GCACACTGACCCAATTAAAG	20	5	ATTAAACATGGTGCGATAGG	20	645	641
rsbC	3320463	*P. mirabilis*	TGGCTTGGAAACACAATC	18	31	AATTCGAGTGATGGACAAAC	20	729	699
speB	31616612	*P. mirabilis*	CGAGCATGACACTAATAATGG	21	606	CCGCTTGCAGATATAGCAT	19	946	341
stmA	3769579	*P. mirabilis*	TACGTACATCGCCACCAG	18	157	AAAGGAACAGGACTACTTTGAG	22	479	323
stmB	3769579	*P. mirabilis*	GAAATACGCATTAGGCTCTG	20	3	GCGTTATAGCAGTTTAATCCA	21	362	360
terA	5702376	*P. mirabilis*	CAAGGTTTCGCTAACTAAAGAG	22	540	ACTTTGATTGATCCGTTTTC	20	1055	516
terD	5702376	*P. mirabilis*	TTCTAAAGGTGGTAATGTTTCTT	23	15	GCATATCCTTGGCCTACA	18	530	516
tna2	150910	*P. vulgaris*	CTAAATATGGCGCAGGAAC	19	197	ATAGTGCCAATCATCCAAAG	20	749	553
uca	881951	*P. mirabilis*	GCACTAGCTACTATTCTTTCTGCT	24	19	AACGGTTGCTTTTACATCC	19	519	501
umoA	3360256	*P. mirabilis*	GAGCCTTGTTTATCCCTCTT	20	11	TCTATCCCAGCGGTTAAAA	19	510	500
umoB	3360259	*P. mirabilis*	GCTCATATCATCTTCCATCC	20	92	CCTAAGCAAATTAACGAACC	20	641	550
umoC	3360262	*P. mirabilis*	AAGATAGGTACGCTTTTAATTTCT	24	4	AGTAATAATTTAGCTCGCCATT	22	404	401
ureDPrmi	150914	*P. mirabilis*	GAGGTACTGCATCGCAAA	18	275	TACCTTCTCTAAATGGTGTTGA	22	657	383
ureEPrmi	150914	*P. mirabilis*	GCGCTTGAACTAACCTCTAC	20	37	GGATGACCCACCATAAGC	18	450	414

gene	gi	source species	forward	length	position	reverse	length	position	product
ureFPrmi	150914	*P. mirabilis*	GCTCAGCAGAAACCTTGTC	19	110	CTGGCGTAAACTGCCAATA	19	609	500
ureR	45657	*P. mirabilis*	TCACAGTCACCACTAATCTCAC	22	122	ACGAAACGCCCTCTTTAC	18	634	513
urg	45933	*P. vulgaris*	GAAAGGACTTAAACTTAACTATCCAG	26	75	AGGGTCGTGAATAGAAACAAG	21	294	220
xerC	2645801	*P. mirabilis*	ACGTCCCTGAAACACTCTC	19	17	AACCAATGCTGTAACCACTC	20	599	583
ygbA	3395515	*P. mirabilis*	CAGGAACGGGTTTCTTATC	19	33	GCTTATAAAAGACTAGATAAATGTCG	26	225	193
zapA	3493594	*P. mirabilis*	GGGATCTTCTATAACTATTCAACC	24	67	TAACCTGGAACATTCTGACC	20	626	560
zapB	3493594	*P. mirabilis*	TTTCTTTGATCTACCTTGGTTC	22	408	TGTAGGCTGCTTTACTACTATCC	23	916	509
zapD	3493594	*P. mirabilis*	AAGAACAAGTAGCAGGTAAAGAG	23	128	CAGTTAGCTTTGCGATGTG	19	703	576
zapE	3493594	*P. mirabilis*	CCAACTTACTTCTATCTACCTGATG	25	28	GTTCCAGAGTATTGGTATTGTTC	23	653	626
algB	150990	*P. aeruginosa*	CACTTTCCGTTATTGCCTC	19	66	GAGGATGAGGATGTTGGC	18	522	457
algN	150999	*P. aeruginosa*	GACTGGCTGAATCGTCTC	18	76	GCAGGTCGTACCAGGAAG	18	364	289
algR	151003	*P. aeruginosa*	ATTGTCGATGACGAACCTC	19	13	TTCAGGTAGAGCTGGAAATG	20	671	659
aprA	45279	*P. aeruginosa*	CATTGAAAGGTCGTAGCG	18	20	CGACGAAGTGGATATTGG	18	364	345
aprE	45279	*P. aeruginosa*	GGTCAAGCACATCCTAGTG	19	186	ACTTCCTTGCGGTACTCC	18	761	576
ctx	45312	*P. aeruginosa*	CCGAAGGACTTGGTTTACT	19	148	CTATGGAAGGTGCCTGTG	18	728	581
ExoS	13892017	*P. aeruginosa*	CGTTTGGGACAGATTGAG	18	67	GATACTCTGCTGACCTCGC	19	695	629
fpvA	1633044	*P. aeruginosa*	AATGCGATAACCATCAGC	18	331	CCGTCGTACTGGAAGTTG	18	662	332
glpR	1399486	*P. aeruginosa*	CAAGCACAACAAGAAATACG	20	9	TAGACCTCCGAAGAGTTGC	19	683	675
lasRa	309873	*P. aeruginosa*	CTGGGACGTTAGTGTCATC	19	96	GTCTTGGCATTGAGTTCG	18	551	456
lasRb	151325	*P. aeruginosa*	GAGCGACCTTGGATTCTC	18	81	ATAAGACCCAAATTAACGGC	20	710	630
lipA	45340	*P. aeruginosa*	AAGAAGTCTCTGCTCCCC	18	7	ACGATTTCCTCCACCTGT	18	281	275
lipH	483463	*P. aeruginosa*	ATGGCAGTTTCAGTGTCG	18	53	CGAAATAGTCGTCCAGCC	18	700	648
Orf159	4545246	*P. aeruginosa*	AATGTCGGCATCATTCTC	18	28	GTAGACCTCGCGCTTGAA	18	432	405
Orf252	4545242	*P. aeruginosa*	GACCTGCTGTTCCAGTTG	18	172	AATTCACGGGTTTTCTCG	18	713	542
OrfX	1399486	*P. aeruginosa*	ATGGATGCTCGGGTACTG	18	97	CTCAGCTACAGCCACGAC	18	420	324
pa0260	15595198	*P. aeruginosa*	GATCGTCTCTGCCCAGTC	18	27	ACATTGATGGTGTCGTCC	18	695	669
pa0572	15595198	*P. aeruginosa*	AGGAGAGAACATGAGTCGC	19	36	TCCTTGTCCCAGTAGTTACC	20	845	810
pa0625	15595198	*P. aeruginosa*	AGGAGCAACTGAAGCGAC	18	236	TCTGCCTTTACCCAGGAC	18	759	524
pa0636	15595198	*P. aeruginosa*	AAGGTTGGCAGGATCAAC	18	76	CTAGTGGCGAAATTGAACAG	20	779	704
pa1046	15595198	*P. aeruginosa*	AGGCATCCATCGAGCTAC	18	386	AACGTCCGAGCAGGATAC	18	619	234
pa1069	15595198	*P. aeruginosa*	GCGAGGAGGTATTCGACA	18	539	CCCTTCTGCGAGTAGTGTT	19	887	349
pa1846	15595198	*P. aeruginosa*	AAGGACTTCTGGTCGGTG	18	286	CAGGAACAGGTGCTCGTAG	19	687	402
pa3866	15595198	*P. aeruginosa*	TTCCCTAACGAATGCTGTC	19	1365	CGTTGCTCCCTCATACAC	18	1878	514
pa4082	15595198	*P. aeruginosa*	CGAGCACCAATATCGAAC	18	1148	GAGCCGTAGGTGTTATCG	18	1751	604
pchG	4325021	*P. aeruginosa*	CCTGCTCAACACCTTCTATC	20	354	GTCGAACAACGCGAACAG	18	780	427
PhzA	5616088	*P. aeruginosa*	GTTGAAAGGGTTTACCGAC	19	18	AATTTCTGCATCGGGTTC	18	434	417
PhzB	5616088	*P. aeruginosa*	ATGCTCGATAATGCTATTCC	20	1	TTCTCGTAGTAACCCTCGG	19	356	356
pilAp	18535593	*P. aeruginosa*	GCTTTACCTTGATCGAACTG	20	17	TCAATAGAGCCAGTCACACC	20	428	412
PilAp2	21629637	*P. aeruginosa*	TGCCGTGAGTGAAATCAG	18	123	CGTAGTTGGCTTTCCAGTT	19	442	320

gene	gi	source species	forward	length	position	reverse	length	position	product
pilC	18535591	*P. aeruginosa*	GGTATCAACCCACTAAAGGTC	21	127	GTCCAGAGCTTCTACCAGAG	20	864	738
PLC	151492	*P. aeruginosa*	GACTTCGCTGTTCGACTTC	19	1356	TCGGTTCGAGTTCATAGC	18	2026	671
plcN	151497	*P. aeruginosa*	GTGTTCCAGGTGTTCGAC	18	1570	GATAGACGTTGTCCTTGACC	20	1873	304
plcR	151499	*P. aeruginosa*	ACAACCTGGAACAGCAACT	19	23	CGACTCTTGCGCGTATTC	18	324	302
PstP	4545246	*P. aeruginosa*	GAAGTGAACTCCGCCAAG	18	31	TCGAGCATCATCAGGTAGAC	20	770	740
purK	1621599	*P. aeruginosa*	TCGAGAAGTCGATGTTCAAG	20	299	CTTGCCGTAGTGATGCAG	18	978	680
pvdD	1633044	*P. aeruginosa*	GTCAAGGGTGTTGTCTGC	18	6665	CTCTGCACAAACTCAGGG	18	7386	722
pvdF	1633044	*P. aeruginosa*	AAGTCGGTACTGGAGTCCTT	20	100	TCAGCACATCATGGAACA	18	715	616
pyocinS1	286179	*P. aeruginosa*	CTTCAGTTCCGAGATGCC	18	241	GTAACGAACGCTATCGGG	18	981	741
pyocinS1im	286179	*P. aeruginosa*	ATATACGGAAAAAGAGTTTCTTGAG	25	21	AGCACGCCATTCTTTAACTTC	21	231	211
pyocinS2	286182	*P. aeruginosa*	TATACGGCTTCAGACTTTCC	20	382	TGGCATAAGTATTGGCAG	18	970	589
pys2	15595198	*P. aeruginosa*	TCGCCAATAAGAAGAAATTG	20	758	AGTGGTACTCGAAGGGTTCT	20	1509	710
pys2	15595198	*P. aeruginosa*	ATCCAGTATATTCCTGCTCG	20	70	TGCAATTTCTTCTTATTGGC	20	779	752
rbf303	836903	*P. aeruginosa*	ATCGTTCTGGTCTTCCTTG	19	157	ACCAAAGAGTGTTGATAGCC	20	895	739
rhlA	452502	*P. aeruginosa*	AGTCTGTTGGTATCGGTTTG	20	13	CTCCAGGTCGAGGAAATG	18	768	756
rhlB	452502	*P. aeruginosa*	AACGCTTTCTCGATCAGG	18	731	GATACTGTGCGGTTGTGA	18	1203	473
rhlR	1117916	*P. aeruginosa*	TTCGATTACTACGCCTATGG	20	118	GGTCCATTGCAGGATCTC	18	576	459
TnAP41	216903	*P. aeruginosa*	CCCCTATTCCATACCTTACC	20	86	TATTGAGGACGCTGATTTTC	20	604	519
toxA	15595198	*P. aeruginosa*	GTGCGCTACAGCTACACG	18	328	CTTGCCTTCCCAGGTATC	18	744	417
uvrDII	3249556	*P. aeruginosa*	AGACCTACAACAAGGTTTCG	20	281	TGAGGATAGTCCCTTCGC	18	490	210
vsmI	695153	*P. aeruginosa*	ATTCCTCTCTGAATCGCTG	19	9	AATATCTTCATCGCCAGTTG	20	359	351
vsmR	695153	*P. aeruginosa*	AGGAATGACGGAGGCTTT	18	4	GGGTTGGACATCAGCATC	18	533	530
xcpX	45433	*P. aeruginosa*	TTCAACCTCAACGGACTG	18	328	TGCAAGGTACTCACCAGC	18	896	569
5triphosphatase	3786332	*C. albicans*	GACACTCCGTCAGATTCAAC	20	148	ATTCCCGTCATCAACATTAG	20	684	537
AAF1	1177214	*C. albicans*	ACATAGTCAGCCACAACCAC	20	564	GCACTCGAATTGTTATTTCC	20	1124	561
ADH1	608689	*C. albicans*	TCAAGAAAGCTACTGATGGTG	21	701	ATTTACTGGTGTCCAAGACG	20	1051	351
ALS1	704426	*C. albicans*	CTCAGTCCTTTGCTACAACC	20	1334	AACGGTATCAGTTTCACCAG	20	2040	707
ALS7	9754770	*C. albicans*	AACGGTCATATCCAAAGAAG	20	42	ACCCATGAAACAAGGTGTAG	20	627	586
ARG56	1770097	*C. albicans*	AAACGCATTGTTAAGAGACC	20	1068	ACCTCTAGCACCAATCAAAG	20	1629	562
ASL43f	3598683	*C. albicans*	CAGGTAAGTCAAAGTCTGGTG	21	476	ATTGCCACCTTGTATATTGG	20	999	524
BGL2	532775	*C. albicans*	GATTAATGACATCAAGGGTTTAGT	24	402	GCAAAGCTTGCATAACATC	19	631	230
CACHS3	218361	*C. albicans*	TCTGGTGAAGGTTTAGGAAG	20	61	ACCACTTGACTCGAACAAAC	20	692	632
CCN1	510578	*C. albicans*	TCGACCATACCATCCAATAC	20	69	TTCACAAAGAAACCTAGCAAC	21	681	613
CCT8	3419680	*C. albicans*	CACCAAACTCAGGCTTATTC	20	23	AGACCATACCCTTGACCAC	19	655	633
CDC28	520627	*C. albicans*	AGAAAATTTGGCCTGATGTTA	20	690	GGTGTTTGTCAATCCCAATA	20	940	251
CDC37	18029900	*C. albicans*	GAATGCAAAGAAACATTGAAA	21	125	CCGTATGCAAATCATCACT	19	589	465
CEF3	2497	*C. albicans*	TGTCTGCTGCTAGTGAATCC	20	2	AAGCAATTTGAGCTTTAGCA	20	505	504
CHS1	2501	*C. albicans*	TGACAGGTTCATTGGTGTC	19	1412	ACAGCCTCAAATTCTCCAC	19	1937	526
CHS2	170844	*C. albicans*	TCAGATGGTGATGAACTGTC	20	76	GACTTCTTGTTGTGGAGGAG	20	696	621

gene	gi	source species	forward	length	position	reverse	length	position	product
CHS4	2073104	*C. albicans*	AACTAGGGCTGCTAATGTTG	20	1434	TTGAGCAAGAGATCCTGAA	19	2040	607
CHS5	3551164	*C. albicans*	AGTCAGAGCAGGGTCAATC	19	108	ATGTCGGGTAAGACACAATC	20	800	693
CHT1	15530177	*C. albicans*	GTCAACAACAAGGCAAGAC	19	260	CTAGCCACGACTCTTTGAAC	20	770	511
CHT2	571426	*C. albicans*	CTGTCAAGAAACTGACGTTG	20	129	TTGTTGTAGAATTGGATGAATG	22	638	510
CHT4	11496138	*C. albicans*	TTGGCTCGATTAAGAAATAAA	21	496	CTGAAACATCACCAGCAGA	19	1072	577
CLN2	510580	*C. albicans*	AACAGCAACCAGAAATCAAG	20	191	TTAAATCAACAGGAATCTTCAAC	23	754	564
CPH1	558949	*C. albicans*	GCTACCACTTTAACCGACAC	20	643	CTTCTCCACCATTAGCACTC	20	1222	580
CSA1	3406797	*C. albicans*	GCTCCATCTAGCAACTCATC	20	2695	GAGACCAACAAACACGAAAC	20	3603	909
CYB1	1103927	*C. albicans*	TTTCATCACCACCTCAAGTC	20	140	ATTTCAACAAGCCAATCAAC	20	743	604
EDT1	25989656	*C. albicans*	AACATTAGAAACGGAACAGG	20	28	CTCAGTTCTGCATTTGGTTC	20	719	692
EFG1	6468469	*C. albicans*	CCAGCAAACAATTCCTAATC	20	342	TTCCCATTACCGCTAGAAC	19	1076	735
ELF	2707896	*C. albicans*	CAAAGTTCCACCATTTCAAC	20	292	ATTGATCCTAATGCTTCCAC	20	797	506
ESS1	12655855	*C. albicans*	ATCGACATCAACAGGCTTAC	20	6	TCTTTGGAGGATATGGACAC	20	525	520
FAL1	6468269	*C. albicans*	CAATAGCACAGGCACAATC	19	197	AAATCTCATCCCTCTTCACC	20	703	507
GAP1	18568389	*C. albicans*	TTCATTCAAACCAGCCTTAC	20	102	TTCACCATACCATCTTACACC	21	636	535
GNA1	4115734	*C. albicans*	CACAAGGTTATACATTCAGAAAAC	24	11	CTACATACCATTTCAACACCAC	22	443	433
GSC1	2274846	*C. albicans*	AAACCATAAATCAACAACCAC	21	1448	CACCAACACATCTCATTGTC	20	1987	540
GSL1	2274848	*C. albicans*	TATTATGGCGATTCCACAG	19	724	CAGCCCAAATTCTTGTTC	18	1297	574
HIS1	4033356	*C. albicans*	CGTTTGTTATTTGCTGTTCC	20	28	GCTGCTTTCATAGTTTCACC	20	533	506
HTS1	21724236	*C. albicans*	AGCTAAATCCAAAGACGATG	20	249	CCTTGATTATGCGAGAAATG	20	787	539
HWP1	4028879	*C. albicans*	GCTACCACTCCAAACACTTC	20	709	AGACGACAGCACTAGATTCC	20	1738	1030
HYR1	11121427	*C. albicans*	TTGGTGGGTTAGAAGTTGAG	20	176	TTGAAACAGCATGAACAATC	20	703	528
INT1a	1144530	*C. albicans*	CAAGAGAAAGGGAAAGAAGAG	21	184	AGGTTTCGTCGTATGAAGTG	20	711	528
KRE15f	170875	*C. albicans*	CGTTAACTCAGTCATATACTACATTTT	27	6	GCAATAAAAGAAACCCCAAT	20	365	360
KRE6	1944423	*C. albicans*	GATCCAGATGCTGTAACCAC	20	1171	CATCGTAAATAACCCGTTTC	20	1871	701
KRE9	3435197	*C. albicans*	TAATTTCCCTTGTTGTTTCC	20	23	TCAATCCAACCATCTTCTTC	20	816	794
MIG1	4688626	*C. albicans*	ACATTCATTGGGTTCATCTC	20	903	ATTGGTGGTAATTGTGTTCC	20	1556	654
MLS1	6980008	*C. albicans*	TGTCCCAGAAAGTGCTAAAC	20	60	AATACCACCGGAAACTGG	18	588	529
MNT1	1480085	*C. albicans*	GCTCGTTTGATTAGATTTGG	20	22	TGTTCACGGACTAAAGCAG	19	584	563
MP65	12057030	*C. albicans*	TTAGCTCATCAACATCATCAAC	22	58	GCTTTAATTTGACTTGGAGTG	21	704	647
NDE1	7270979	*C. albicans*	ACTGTCGTTTCTGGTCATTC	20	574	ACGAGTGAAATCTCTTGGTG	20	1209	636
PFK2	4490768	*C. albicans*	GGTACGAACAGACAAACACC	20	1921	AACAGCTCTAATGGCAAATC	20	2586	666
PHR1	8132881	*C. albicans*	TTAGCCAAGTTTGAATCGTC	20	55	AATTTCTTCGTCATCATTGG	20	642	588
PHR2	2293529	*C. albicans*	TCAATCTTGGCTGCTACTTC	20	25	GGCTAATGAAACTCTGATGG	20	633	609
PHR3	6911252	*C. albicans*	ATTGGGTATCAACACCATTC	20	114	CCATAGTTGTTTGGTTCCTG	20	797	684
PRA1	1916851	*C. albicans*	ATTAGCTGAACATGCCAGAG	20	192	ATCTCCATTGCACCCTTC	18	723	532
PRS1	1276446	*C. albicans*	GATTTACACGCCTCACAAA	19	391	GCAATAACAGCAGAAATATCAA	22	896	506
RBF1	1378025	*C. albicans*	AACAGCAAGCTGCTCAGT	18	1082	TTACAAAAACCCACTTCTTTTC	22	1584	503
RBF1	1378025	*C. albicans*	CCCAACTAATTCAGCATCAC	20	36	GTACCAATTCGGCTTTACTG	20	589	554

gene	gi	source species	forward	length	position	reverse	length	position	product
RBT1	9963981	*C. albicans*	TACCACGATAGCTCCATTTC	20	136	ACCCAAAGTTCTCTTGCTG	19	741	606
RBT4	9963985	*C. albicans*	GTGGTGTTACTGTTGGTGAA	20	83	GTAGCATCCCAAGTCAAATC	20	707	625
RHO1	2766184	*C. albicans*	AAGAGATGATCCTCATACTATTGAA	25	375	CAAGACAACACATTTCTTCTTC	22	594	220
RIM101	5802567	*C. albicans*	GTCCTTTCAAGTGTTTGTGG	20	443	TATTGTTGTTGTGGGTGTTG	20	1166	724
RIM8	5802569	*C. albicans*	ATAACCCACATAAGGTCTGG	20	119	AGCTAGTTCGTGGATCTTTG	20	661	543
RNR1	7271005	*C. albicans*	TAAGAGAGATGGCCGTAAAG	20	12	TTACCGTGAATACCGACAG	19	542	531
RPB7	12655857	*C. albicans*	TTTTGGACCTCAAATGGAC	19	51	AAATAGTCTTCTTTTATGCTTCCT	24	503	453
RPL13	4115901	*C. albicans*	CCAAGAACTTACCATTATTGAAC	23	11	TTTCGTTTCTAGCCAATCTC	20	535	525
RVS167	7271023	*C. albicans*	TTAAAGGATTCAAAAAGGGTGT	22	8	AATAATTCTTGTGCTGGTTCA	21	407	400
SEC14	897694	*C. albicans*	TGACTACGATGACTACTGAAGAA	23	2	TTTTAGCAACAATGGGTTTT	20	316	315
SEC4	2642181	*C. albicans*	ATTGGTTTCAAACAGTTACTCA	22	320	ATTCTCTTCAACATGCTTCTCT	22	549	230
SHA3	7271025	*C. albicans*	CATGGCACCAGAAAGAAC	18	123	AATTGTTGGTTTGCAGTAGG	20	644	522
SKN1	1944425	*C. albicans*	TATAATGCCCCGAAAATAAA	20	19	TTAAGCGGATATCTATCGTACC	22	518	500
SRB1	3777502	*C. albicans*	TGGAACCTGTTTGTACTTGAC	21	684	CTAGCCCACTTTCCAATTC	19	962	279
TCA1	2852981	*C. albicans*	GATTTCCTAGCCGGAATG	18	21	ATTATGTAACCTTAGTCGTAATTGTC	26	617	597
TRP1	3419680	*C. albicans*	AAGACGACTGAGCGTGTC	18	3	TGGACTGGAAGATAAGCTAGAG	22	352	350
TUP1	2245633	*C. albicans*	AAAGAGCTAACCACGTCAAG	20	443	GATCGACAAAGTCAAGGAAC	20	1017	575
YAE1	7271003	*C. albicans*	ACATGTCAAGAGGATTGTTCA	21	4	TTTACATTGATTGATTAAATTTGC	24	333	330
YPT1	15077427	*C. albicans*	AGGTGGTGTCATGAAATTATTAG	23	333	ACAGGAATTCGATTGGTTAG	20	618	286
YRB1	13518294	*C. albicans*	GCCGAAGATACTAAACCAAA	20	7	TCAGAAACATCAGCAGTAACA	21	530	524
YST1exon2	7270972	*C. albicans*	TCTGATGTTGCTGTTTGTTC	20	121	ATCTTCAGTTTCACCAGTCC	20	633	513
ZNF1CZF1	170941	*C. albicans*	TTGCCCAATCAGCATTAC	18	448	ACACCTTGGTCTTGTTTCAC	20	1005	558
abcXStrpmut	2952523	*S. mutans*	GAAATGATATTGACGGGACT	20	41	TCAACACAAGGACAGGTTG	19	621	581
acrA	17973457	*P. mirabilis*	CTGGCTCTGTTAGTGCTTTC	20	28	CTGTTACCGTTGATTTACCTG	21	571	544
acrB	17973457	*P. mirabilis*	CCTTTGAAGCTGGTACTGAC	20	278	CTGTGAACCGTCTTGATTG	19	777	500
acrR	17973457	*P. mirabilis*	TGTCATCATAGCTCTTAACATAATC	25	130	CTAAACGCCAAGCACAAG	18	632	503
albA	22135424	*K. oxytoca*	TGAGCAGCGTAACCAGAC	18	60	AGTCATCCACGTTCCTTTC	19	591	532
arr2	12024948	*E. coli*	GCAAGGACCGTTCTATCA	18	48	CTTCAATGACGTGTAAACCA	20	448	401
cadBStalugd	1658278	*S. lugdunensis*	GAGGTGTAATTATGATTCAGACTATT	26	3	TCGTACCAACTAATTTAGACAATC	24	346	344
elkT-abcA	1841513	*S. aureus*	ATTAGAAATTGCGACTGGTG	20	786	AGCGTGTCATATCCTTCATC	20	1394	609
emeA	22775586	*E. faecalis*	GACCAGGAGTTGGTGGTT	18	464	AACAGCTGACAAATCAACG	19	806	343
mexA	5616092	*P. aeruginosa*	CTCGACCCGATCTACGTC	18	34	GTCTTCACCTCGACACCC	18	536	503
mexB	5616092	*P. aeruginosa*	TGTCGAAGTTTTTCATTGATAG	22	2	AAGGTCACGGTGATGGTC	18	281	280
mexR	886021	*P. aeruginosa*	GAACTACCCCGTGAATCC	18	3	CACTGGTCGAGGAGATGC	18	413	411
mreA	7548683	*S. aureus*	TACGATGACACCAGTCTTTG	20	15	ATCGACAAAACGTACAGGAT	20	756	742
msrCb	6687492	*E. faecium*	AGAACATATCCGCAAACAAG	20	30	TGATGTAAACAAAATCGTTCC	21	399	370
norA23	4115706	*S. aureus*	GGTTACTTGTTGCTGCTTTT	20	122	CGTAATCGCAATCGAAATA	19	735	614
nov	558416	*E. coli*	AACCGTTTATACGTTGGTGAG	21	6	GATTAACGCCTGCATCAC	18	405	400
qacEdelta1	12024948	*E. coli*	AAGGCTGGCTTTTTCTTG	18	5	AATTATGAGCCCCATACCTAC	21	285	281

gene	gi	source species	forward	length	position	reverse	length	position	product
rtn	558808	*P. vulgaris*	CCTGAAATCCACTACTGACC	20	180	CCAACGACAGTTCCACTC	18	752	573
sul	17129524	*E. coli*	CATCGTCAACATAACCTCG	19	27	AATTCTTGCGGTTTCTTTC	19	810	784
sulI	12024948	*E. coli*	CTCTTAGACGCCCTGTCC	18	202	GTTTCCGAGAAGGTGATTG	19	785	584
uvrA	2696019	*E. faecalis*	GCGTAGAACGTGGACTTG	18	86	TCATAAATGGACCGAATACC	20	668	583
wbbl	22759339	*K. pneumoniae*	ATAAAGCAATGAAGCCTAAAGT	22	8	TTTACGATCAAGGAACCATC	20	777	770
wzm	22759339	*K. pneumoniae*	ATGAAAAACCCTCATCAAAA	20	1	GGGAAAAAGACCAAAGGTAA	20	500	500
wzt	22759339	*K. pneumoniae*	ATGTTCTCGCTGCAGTTC	18	722	ACCAAAACTTTATTTTCATCATC	23	1261	540
aacA_aphDStwar	31074829	*S. warneri*	AGTTGAAAATGAAATATGTATAAGAAC	27	9	AACTGCATTAGCATTTCTTTC	21	399	391
aacA4	14276839	*P. aeruginosa*	TGCGATGCTCTATGAGTG	18	18	GTACCTTGCCTCTCAAACC	19	440	423
aacA4	14276839	*P. aeruginosa*	GCTAAATCGATCTCATATCGTC	22	36	GGCGTGTTTGAACCATGTA	19	487	452
aacA-aphD	3676412	*S. aureus*	AGATTTGCCAGAACATGAAT	20	473	TGTTGCATTTAGTCTTTCCA	20	1085	613
aacC1	13094933	*P. aeruginosa*	CCTGACCAAGTCCAATCC	18	31	TAGATCACGTAAGCACCAAG	20	359	329
aacC1	13094933	*P. aeruginosa*	TCATTCGCACATGTAGGC	18	8	GGGATCGTCACCGTAATC	18	387	380
aacC2	45769	*E. coli*	GACCGATCACCCTACGAG	18	184	CGAAATGCTTCTCAAGATAGG	21	799	616
aadA	3540256	*E. faecalis*	GCCGAAGTATCGACTCAAC	19	19	AGCACTACATTTCGCTCATC	20	563	545
aadB	623029	*E. coli*	GCAGGTCACATTGATACACA	20	60	CATATCGCGACCTGAAAG	18	574	515
aadD	21623792	*S. aureus*	GCTATTGGTGTTTATGGCTC	20	97	CTGATTGCTTAACTGCTTCA	20	598	502
aphA3	3138990	*synthetic construct*	AAGATACGGAAGGAATGTCTC	21	124	ACAAAGATGTTGCTGTCTCC	20	655	532
aph-A3	1272325	*S. aureus*	GAGAATATCACCGGAATTGA	20	12	GCTCGACATACTGTTCTTCC	20	718	707
strA	17222205	*E. coli*	CTCATTTGGCTCAAAGGTC	19	163	TGAATAGGACAGCGAAGG	18	727	565
strB	17129524	*E. coli*	AAGTTTCATTGCCAGACG	18	86	TAGACTGCGTTGCTCCTC	18	792	707
ampC	28864526	*K. oxytoca*	TTATCTGCAACACTGATTTCC	21	13	TTTCCGGCACAGTAATAAAG	20	637	625
bla_FOX-3	1841437	*K. oxytoca*	CTTACTATCGGAGCTGGTCA	20	461	AGGGCATCTCCCTGATAC	18	800	340
blaA	522103	*P. vulgaris*	TCACTCATTAACCATTGCTG	20	26	GTATGTCACTCAAGGTGCTG	20	793	768
blaB	522103	*P. vulgaris*	ACACTGGCTGAATTAAGTGC	20	367	AACCCGCTTTAATACTGTTATC	22	688	322
bla-CTX-M-22	19526203	*K. pneumoniae*	TCACGCTGTTGTTAGGAAG	19	50	AGGCTGGGTGAAGTAAGTG	19	807	758
blaIMP-7	13094933	*P. aeruginosa*	ACTGACGCTTATTTGATTGAC	21	178	GTACGTTTCAAGAGTGATGC	20	680	503
blaIMP-7	13094933	*P. aeruginosa*	TTGACGAAGGCGTTTATG	18	89	TGTGACTTGGAACAACCAG	19	646	558
blaIShaemolyt	8574410	*S. haemolyticus*	GCCAATAAGCAAGTTGAAATA	21	4	ATTTAAAATGTCTCGCAATTCT	22	363	360
blaOXA-1	152062	*Plasmid RGN238*	TTTGAAGGAACTGAAGGTTG	20	109	TTCTATTTGCTGTGAATCCTG	21	673	565
blaOXA-10	12024948	*P. aeruginosa*	TAGCTCGTGCATCAAAGG	18	167	AACCTCTGTCTCCTTCTCAAC	21	696	530
blaOXA-2	47874	*S. typhimurium*	CACGATAGTTGTGGCAGAC	19	126	ACTCAACCCATCCTACCC	18	676	551
blaOXA-32	14276839	*P. aeruginosa*	ACGTTCTGACTGGAGGAAG	19	81	TTCAATAGAGCGAAGGATTG	20	783	703
blaOXY	18844705	*K. oxytoca*	GACAATACCGCGATGAATA	19	400	CCGGTGGTATTGCCTTTTA	19	659	260
blaOXY-KLOX	32130556	*K. oxytoca*	TGTGCAGTACCAGTAAGGTG	20	385	CTCCGGTTGGGTAAAGTAG	19	980	595
blaPER-1	396144	*P. aeruginosa*	CCATCAGGCAACAGAATG	18	137	TGACGATCTGGAACCTTTAC	20	769	633
blaPrmi	7672820	*P. mirabilis*	GCGGTAAGATCCTTGAGAG	19	152	AGTGGTCCTGCAACTTTATC	20	653	502
blaRShaemolyt	8574410	*S. haemolyticus*	AGCTTTTGTTTTATATTTCTATTGG	25	31	TTTAGTCTTTTGTCAATTACACTTTT	26	587	557
blaSHV-1	4337047	*K. pneumoniae*	CGTAGGCATGATAGAAATGG	20	117	GTATCCCGCAGATAAATCAC	20	785	669

gene	gi	source species	forward	length	position	reverse	length	position	product
blaTEM-106	21464484	*E. coli*	ACATCGAACTGGATCTCAAC	20	131	TCTCAGCGATCTGTCTATTTC	21	832	702
blavim	5420397	*P. aeruginosa*	CATGCGTGTAAATCATCGT	19	72	CCCTTGAGCGGAAGTATC	18	371	300
blaZ	1575124	*S. aureus*	TGCTTTAGTTTTAAGTGCATGT	22	30	TCCTTCATTACACTCTTGGC	20	839	810
cumA	511055	*P. vulgaris*	ACAACATTTCGCCAAACA	18	16	TTGAGCTAAAGACATTCCTGT	21	378	363
dacCStrpyog	1420857	*S. pyogenes*	AATCATCCTCGTGGCTTT	18	150	GCTAAATTGGGATAGTCCTG	20	754	605
femA	4929298	*S. aureus*	TACAGTCATTTCACGCAAAC	20	58	TCACGCTCTTCATTTAGTTCT	21	770	713
femBShaemolyt	4539615	*S. haemolyticus*	AATGATGGCTTTGAAGTAGTG	21	106	CCTGTTCTTGCTTCTGTTTC	20	650	545
fmhA	4574232	*S. aureus*	TGACTTCGGATGAGTTCAAT	20	17	GCTGTTAATTGTTGTTGCTTT	21	779	763
fmhB	4574234	*S. aureus*	CTCACCCAAATGGAGATTTA	20	50	CTTGCTTTTCAGATGTTTCC	20	799	750
fox-6	14626421	*K. pneumoniae*	GGCTTACGGGATCAAGAC	18	723	ATTGGCCTGGAAGCTCAC	18	948	226
ftsWEF	4633279	*E. faecium*	ACAGTGCCAGTTCTTATCGT	20	35	GAAGCCAACCTTTATTTCCT	20	592	558
mecA	13785452	*S. aureus*	AGTTGTAGTTGTCGGGTTTG	20	36	TGAAGTCGCTTTTCCTAGAG	20	750	715
mecISepid	46995	*S. epidermidis*	ATGGATAATAAAACGTATGAA	21	1	CAATTCTTCTATTTCATCTTGTG	23	348	348
mecR1Sepid	46995	*S. epidermidis*	AAGTATAATCAGTTCATTGCTCAC	24	24	GTTTCGACTACGACAGTTGG	20	575	552
pbp1a	23451740	*S. pneumoniae*	CTTGGTTAGCGATTCAGTTAG	21	248	CAATTCATCGTCTGGATAGG	20	777	530
pbp2aStrpneu	6165959	*S. pneumoniae*	CGGAACTGTATAATCCCTTG	20	740	AGTGGCAACAGCAGGTAG	18	1419	680
pbp2b	49391	*S. pneumoniae*	TGAGGAAGGTAGTAAGGGAAA	21	38	CAAACATATTGGGTTGATAGG	21	537	500
pbp2primeSepid	46993	*S. epidermidis*	TAGCAATACAATCGCACATAC	21	915	TGGTATATCTTCACCAACACC	21	1479	565
pbp2x	886954	*S. pneumoniae*	TGAAGATGGCAGCAAGAG	18	368	TCAAACCCTCATTAACATCC	20	891	524
pbp3Saureuc	6912038	*S. aureus*	AGAGATGAATGCAGGAACAG	20	558	ACCACCTTGGAAATGTAATG	20	1272	715
pbp4	7688169	*E. faecalis*	CAAACAAGAATTAGCCGAAG	20	120	CAGAAGCAACTGTGATTGG	19	742	623
pbp5Efaecium	13786076	*E. faecium*	TACAGATGCAGACGGTGTAG	20	963	CCCTGTCCATATCCAGTATC	20	1622	660
pbpC	2149899	*E. faecalis*	GCCGGTGTATCACTAAAGG	19	160	CTGTTCTAGTCCCATTGCTC	20	681	522
psrb	790434	*E. faecium*	TTATCTGTTTTGTTACTGCTTACAC	25	25	TTCTAATCCTCCCAAAGTCC	20	592	568
cat	46651	*S. aureus*	AGAAAATTGGGATAGAAAAGAA	22	88	CTGCAAGGCAACTGGTAT	18	622	535
catEfaecium	7595743	*E. faecium*	GATAACCATCACAAACAGAATG	22	69	TTATTTGAACCAACAAACGAC	21	598	530
cmlA5	12024948	*E. coli*	TGGAATGGGTAGCTTCTTC	19	672	GGGAAACACAAGACAGACC	19	1177	506
ble	1567207	*S. aureus*	TACAGTCTATCCGGGCATT	19	5	TGTTGTCGGGATCAATTACT	20	364	360
ddl	460079	*E. faecalis*	GAAGATGGAACAATTCAAGG	20	307	TTTCCCACAGTAAAGGATACA	21	979	673
vanA	43335	*E. faecium*	GTGCGGTATTGGGAAACA	18	650	CGGCCGTTATCTTGTAAA	18	899	250
vanB	148323	*E. faecalis*	TTGTCTGGTATCCCCTATGT	20	11	AAAAGATCAACACGAGCAAG	20	552	542
vanC-2	1857221	*E. flavescens*	AAGCTGCCTTATGTAGGTTG	20	5	AGCACCGACAGTCAAAGA	18	364	360
vanH(tn)	155036	*E. faecium*	TCTTATGGCAGTACGCAAC	19	345	CCAGTTTCCCGTTTTCTAAT	20	748	404
vanHB2	11878534	*E. faecium*	AAGTGTGGGCATTACTGTTT	20	9	CCTTGCTTCATCTCTCCA	18	671	663
vanR	148331	*E. faecium*	ACGAGAATTATACGGTTTTCA	21	71	ATCAATGGTGTCGTTCATTT	20	642	572
vanRB2	11878534	*E. faecium*	GAATTCTACTTGTCGAGGATG	21	11	TCTGCCTCTGCATCAAAC	18	290	280
vanS(tn)	155036	*E. faecium*	AATGATCCGAGGGAAACT	18	117	TTTGTTAGCGTTATCGTTTGT	21	680	564
vanSB2	11878534	*E. faecium*	ACATGAGTTGGAGGAAACAC	20	651	TACGATGGCAAGTCCTAAAC	20	1245	595
vanWB2	11878534	*E. faecium*	GGCAGCAAAGACCTTAAAC	19	231	TCATTGGTTCTCCTCCTG	18	828	598

gene	gi	source species	forward	length	position	reverse	length	position	product
vanX(tn)	155036	*E. faecium*	GACATACGAGTTGGCTGAA	19	126	CTATTGGGGTATGGTTCGT	19	584	459
vanXB2	11878534	*E. faecium*	TGGGATAACTTCACAGGAAA	20	70	GGTATGGCTCATCAATCAA	19	577	508
vanY(tn)	155036	*E. faecium*	ATACTTAGGTTATGACTACGTTAATGA	27	42	AAATGAACCCGTATTTCCA	19	592	551
vanYB2	11878534	*E. faecium*	GTGCGTTCATTATTTCGTTC	20	83	GCCATCTATAAACCTCGTTG	20	634	552
vanZ(tn)	155036	*E. faecium*	AGGATTGCTAGCTTTATATTTAGTG	25	21	ATTTATTCTTAAATGGGTACGG	22	477	457
ermA	13785452	*S. aureus*	CCAGAAAAACCCTAAAGACA	20	6	AAAGAACACGATATTCACGG	20	619	614
ermB	6273677	*S. aureus*	CATTTAACGACGAAACTGG	19	124	AATTGTTTACTTTGGCGTGT	20	667	544
ermC	4138444	*S. aureus*	ACACAGTCAAAACTTTATTACTTCA	25	21	CAACAAGTTTATTTTCTGTAGTTT	24	220	200
linB	4633124	*E. faecium*	CGTTTACCAAAGGAGAAGGT	20	86	TGTTTAGCCAATTATCAGCA	20	595	510
mdrSA	3892641	*S. aureus*	GACAGATTTTCGATCCCTTA	20	45	CCTTTTTGTTTTGATGCACT	20	752	708
mefA	18478326	*S. pyogenes*	GGTCTTGTCTATGGCTTCAC	20	132	CATTCCTTCTTGCATTTCTC	20	636	505
mphBM	3892641	*S. aureus*	GCATATAAATATCAAAACCATACAAG	26	429	TTTCAATGCCATCGTTTC	18	879	451
mrx	807701	*E. coli*	GTTGAGAATGGGAGAGACTG	20	259	AACGTCGCGATTCTAGGT	18	798	540
msrA	15077570	*S. aureus*	CATTAGCAGGAGGATGTTTC	20	20	TGAACCACGTTGATATTGG	19	486	467
satA	433714	*E. faecium*	GTCCGAATCCTATGAAAATG	20	23	GCAGCTGATTTATTGTATCTTG	22	547	525
satSA	1272325	*S. aureus*	GTGATTACAGAAATGAAAGCAG	22	1	AGATATATTACCTTATCAGTATTGTCA	27	227	227
tetAJ	4104704	*P. mirabilis*	TTAGCACTCTATGCGACGA	19	139	AATACACCAAGAAATGCAAGA	21	770	632
tetL	1041816	*E. faecalis*	CACCTGCGAGTACAAACTG	19	140	CCCACAAAGAACTCCAATC	19	795	656
tetM	1065723	*E. faecalis*	GACAAAGGTACAACGAGGAC	20	109	GCACTTCCATGATAAAGAGG	20	662	554
dfrA	3676404	*S. aureus*	CAATTACCTTGGCACTTACC	20	58	CCCTTTTCTACGCACTAAAT	20	480	423
dfrStrpneu	3513549	*S. pneumoniae*	ACTAAGAAAATCGTAGCTATTTGG	24	4	ACTTCCTTTCTCTTGCGATA	20	503	500
