# Supplementary material for: Large scale multiplex PCR improves pathogen detection by DNA microarrays
Source: BMC Microbiol. 2009 Jan 3;9:1. doi: 10.1186/1471-2180-9-1 (PMC2631447; doi:10.1186/1471-2180-9-1)
Supplement: Additional file 2 — Prototype DNA microarray for detection of common pathogens. The figure represents the analysis of microarray hybridizations with decreasing amounts of bacterial DNA. [file 1471-2180-9-1-S2.pdf]

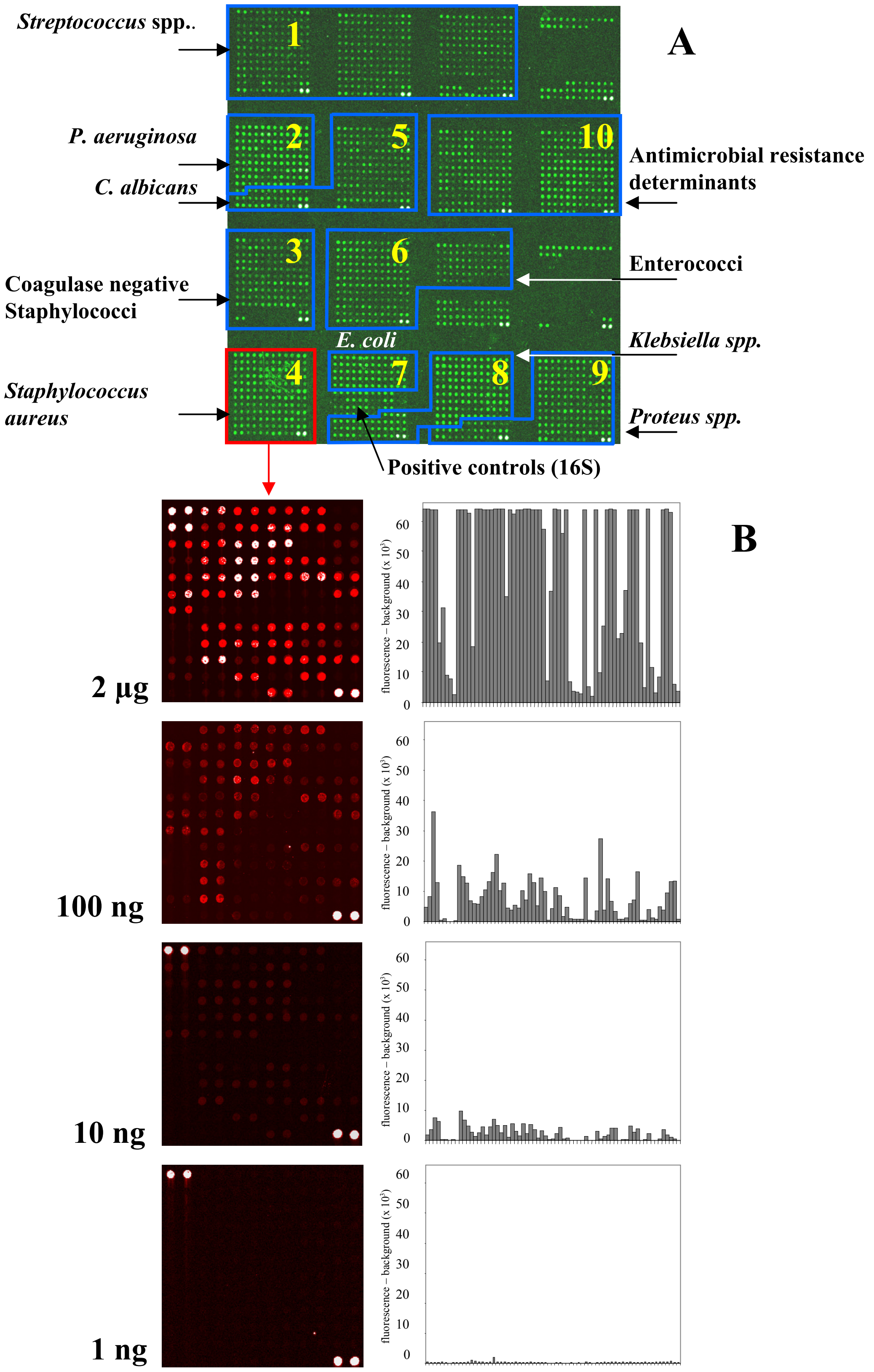

Supplemental Figure 1. (A) Illustration of the DNA microarray prototype established for detection and characterization of pathogens causing sepsis hybridized with Cy3-labelled nonamers. Placement of capture probes corresponded to groups of the most important pathogens (1) Streptococci, (2) *Pseudomonas aeruginosa*, (3) Coagulase Negative Staphyloococci, (4) *Staphylococcus aureus*, (5) *Candida albicans*, (6) Enterococci, (7) *Escherichia coli*, (8) *Klebsiella* spp, and (9) *Proteus* spp. Region 10 contains capture probes for antimicrobial resistance determinants. **(B)** Hybridizations of the microarray with decreasing amounts of genomic DNA of *S. aureus* (ATCC 29213) (2 µg, 100 ng, 10 ng and 1 ng). Raw scans of *S. aureus*-specific region 4 (left) and corresponding quantification of the hybridization signal of the individual spots (right).
